# A No-Reference Multivariate Gaussian-Based Spectral Distortion Index for Pansharpened Images

**DOI:** 10.3390/s26031002

**Published:** 2026-02-03

**Authors:** Bishr Omer Abdelrahman Adam, Xu Li, Jingying Wu, Xiankun Hao

**Affiliations:** School of Electronics and Information, Northwestern Polytechnical University, Xi’an 710129, China; 1845346229@bledu.net.cn (J.W.); haoxk@chinatelecom.cn (X.H.)

**Keywords:** pansharpening, spectral distortion, no-reference quality assessment, multivariate gaussian model, Benford’s Law, remote sensing images

## Abstract

Pansharpening is a fundamental image fusion technique used to enhance the spatial resolution of remote sensing imagery; however, it inevitably introduces spectral distortions that compromise the reliability of downstream analyses. Existing no-reference (NR) quality assessment methods often fail to exclusively isolate these spectral errors from spatial artifacts or lack sensitivity to specific radiometric inconsistencies. To address this gap, this paper proposes a novel No-Reference Multivariate Gaussian-based Spectral Distortion Index (MVG-SDI) specifically designed for pansharpened images. The methodology extracts a hybrid feature set, combining First Digit Distribution (FDD) features derived from Benford’s Law in the hyperspherical color space (HCS) and Color Moment (CM) features. These features are then used to fit Multivariate Gaussian (MVG) models to both the original multispectral and fused images, with spectral distortion quantified via the Mahalanobis distance between their statistical parameters. Experiments on the NBU dataset showed that the MVG-SDI correlates more strongly with standard full-reference benchmarks (such as SAM and CC) than existing NR methods like QNR. Tests with simulated distortions confirmed that the proposed index remains stable and accurate even when facing specific spectral degradations like hue shifts or saturation changes.

## 1. Introduction

Satellite systems face inherent limitations in imaging, storage, and data transmission, leading to a trade-off between spectral and spatial resolutions in remote sensing images [1,2]. Despite technological progress, spaceborne sensors typically capture high spatial resolution (HR) panchromatic (PAN) images alongside low spatial resolution (LR) multispectral (MS) images, rather than direct HR MS data.

Pansharpening, a fundamental image fusion technique in remote sensing, addresses this by combining the high spatial resolution of PAN images with the rich spectral information of MS images to produce high spatial resolution multispectral (HRMS) products [3]. This process enhances the interpretability and utility of satellite imagery, enabling applications such as land cover classification, urban planning, environmental monitoring, disaster management, precision agriculture, visual analysis, change detection, and mapping. The pansharpening field has evolved through key phases since the 1980s [4]: early techniques like Intensity-Hue-Saturation (IHS) and High-Pass Filtering (HPF) [5]; 1990s advancements in Multiresolution Analysis (MRA) [6,7]; and post-2000 innovations including variational optimization (VO) [8,9], deep learning (DL) [10], and unified frameworks [11,12]. Recent advancements in this area have further refined these deep learning approaches by exploring interactions between the spatial and frequency domains to improve feature extraction and fusion accuracy [13].

However, the fusion process frequently introduces artifacts, with spectral distortion being one of the most critical and pervasive issues. Spectral distortion refers to the alteration of the original spectral properties in the fused image, manifesting as color shifts, radiometric inconsistencies, or loss of spectral fidelity. Visually, this may appear as unnatural hues or brightness variations, but its impact extends far beyond aesthetics. In quantitative remote sensing tasks, such as vegetation index calculation (e.g., NDVI), mineral mapping, or change detection, precise spectral signatures are paramount. Even subtle distortions can lead to erroneous interpretations, compromising the reliability of downstream analyses. For instance, altered spectral bands may misrepresent vegetation health or soil composition, leading to inaccurate decision-making in fields like agriculture or ecology.

Despite advances in pansharpening algorithms, categorized broadly into component substitution (CS), MRA, VO, and machine learning (ML) approaches, spectral distortion remains a persistent challenge, exacerbated by factors such as sensor misalignment, atmospheric effects, or algorithmic assumptions about spectral–spatial relationships. CS and MRA represent traditional methods, differing in spatial detail extraction: CS uses spectral transforms to separate and substitute intensity components with histogram-matched PAN data; MRA employs multiscale decompositions (e.g., à trous wavelet or generalized Laplacian pyramid) to inject high-frequency PAN details. VO recasts fusion as an optimization problem with fidelity and regularization terms, drawing from super-resolution and restoration techniques. ML, especially convolutional neural networks (CNNs), excels at managing non-linearities, balancing spatial and spectral quality in satellite imagery.

Quality assessment (QA) in pansharpening is vital for ensuring image reliability and accuracy [14]. It guides fusion algorithm selection, informs new method development, sets standards for downstream applications (e.g., classification or detection), and boosts the commercial appeal of fused products. Effective QA mitigates issues from spectral–spatial trade-offs, preventing distortions that could compromise results. Assessment approaches fall into three main types: qualitative (visual inspection) [15], application-based (task performance, e.g., classification) [16], and quantitative. The latter is considered the most objective, targeting spectral distortion (color alterations) and spatial distortion (artifact introduction or detail mismanagement).

These are subdivided into full-reference (FR) and no-reference (NR) categories. FR QA usually relies on Wald’s protocol [14], emphasizing consistency (degraded fused image matches original LR MS) and synthesis (fused image mimics hypothetical HR MS capture). Reduced-resolution (RR) assessment applies this by downscaling inputs, using the original LR MS as a reference, with filters matching sensor modulation transfer function (MTF) [17]. FR metrics, such as the Spectral Angle Mapper (SAM), Spectral Information Divergence (SID), and Cross-Correlation (CC), require a high-resolution ground truth image for comparison, which is often unavailable in real-world scenarios. However, RR suffers from scale-invariance failures at high ratios, filter biases, and MTF instability from sensor aging.

In contrast, NR methods evaluate the HR MS image directly, avoiding such assumptions, though protocols like Quality with No Reference (QNR) [18] and its variants (e.g., FQNR and MQNR) face criticism for spectral–spatial coupling and a lack of standardization [19]. Traditional no-reference image quality assessment (NR-IQA) or blind IQA includes opinion-aware methods (e.g., BIQI [20], DIIVINE [21], BRISQUE [22], BLIINDS-II [23]) trained on distorted natural images with subjective scores, limiting generalization for pansharpening. Opinion-unaware approaches, like NIQE [24] and IL-NIQE [25], fit features to Multivariate Gaussian (MVG) models, measuring distances from pristine benchmarks promising for remote sensing adaptation. For pansharpening-specific NR QA, evaluations occur at the PAN scale without HR MS references, using innovative distortion measures. QNR-like indices [26] assess band relationships via the Universal Image Quality Index (UIQI), with variants like hybrid QNR (HQNR) [27] and regression-based QNR (RQNR) [28] incorporating MTF and consistency for spectral fidelity. Quality Estimation by Fitting (QEF) [15] extrapolates RR metrics, enhanced by Kalman Filter-based (KQEF) [29] and Combiner-based (CQEF) [19] versions, but these depend on accurate down-sampling, suffer scale-invariance issues, and show uneven performance across scenes and distortions. DL-based NR-QA advances include CNN architectures like the Deep Feature Similarity Measure Network (DFSM-net) [30] and the Three-Branch Neural Network (TBN-PSI) [31], which learn distortions without hand-crafted features, improving correlations but requiring large datasets and computational resources, reducing interpretability. MVG-based NR methods extract features (e.g., NDVI, NDWI, ASM, CON) from pristine MS images to train benchmark models, then compute distances for test images [32,33]. While effective, these often produce global scores, conflating spatial and spectral distortions.

The existing literature highlights several limitations in current NR spectral QA methods. For example, QNR-based indices assume that spectral relationships remain consistent across resolutions, ignoring the non-stationary nature of remote sensing imagery, which includes diverse land covers like vegetation, water bodies, and urban areas. Other MVG-based models, while effective for general image quality assessment, do not incorporate features tailored to spectral artifacts, such as deviations from natural statistical distributions or Color Moments. Our previous studies on datasets like the Ningbo University (NBU) pansharpening database [34] comprising imagery from sensors such as IKONOS (IK), WorldView-2 (WV-2), WorldView-3 (WV-3), and WorldView-4 (WV-4) reveal that pristine MS images adhere to statistical laws like Benford’s Law in their First Digit Distributions (FDDs) within hyperspherical color domains (HCDs), but fused images deviate markedly due to spectral alterations. Simulated distortions (e.g., hue shifts, saturation changes, non-linear intensity mismatches) further amplify these deviations, underscoring the need for metrics sensitive to such changes. Despite these insights, no dedicated NR metric exists that exclusively isolates spectral distortion while leveraging a comprehensive statistical model to capture both local and global spectral characteristics.

Recently, a spectral quality assessment method based on Benford’s Law was proposed, showing a strong correlation with visual perception [35]. Expanding on this concept, a subsequent study developed an NR metric specifically for fused hyperspectral imagery [36]. The findings from this later work indicate that the technique offers superior stability and robustness compared to alternative NR metrics, yielding results that align more closely with full-reference benchmarks.

Various other NR techniques employ a Multivariate Gaussian (MVG) model trained on features extracted from pristine, undistorted images [31,32]. However, these MVG-driven approaches typically generate a single aggregate quality score, failing to distinguish between spatial and spectral distortions.

Despite these strides, methods based on MVG, DL, and sparse coding frequently struggle to generalize across diverse sensors and scenes. Traditional FR protocols rest on flawed assumptions, existing NR metrics like QNR tend to couple distinct distortion types, and DL approaches demand prohibitively large datasets. These limitations highlight a critical need for advanced NR frameworks capable of isolating spectral distortion through specialized statistical features, which serves as the primary motivation for this paper.

To address this gap, this paper proposes a novel No-Reference Multivariate Gaussian-based Spectral Distortion Index (MVG-SDI) specifically designed for pansharpened images. Building on the MVG framework, the method extracts a hybrid feature set from non-overlapping image patches: 9-dimensional FDD features derived from Benford’s Law in the hyperspherical color space (HCS) to detect statistical deviations in angular components, and 12-dimensional Color Moment (CM) features (mean, standard deviation, and skewness across RGB-NIR channels) to quantify perceptual color shifts. These features are concatenated into a 21-dimensional vector per patch, forming a spectral feature matrix. Separate MVG models are fitted to the original MS (reference) and fused (test) images, with spectral distortion quantified via the Mahalanobis distance between their parameters. This approach ensures sensitivity to localized distortions while accounting for feature interdependencies, outperforming existing NR metrics in isolating spectral errors without confounding them with spatial artifacts.

The contributions of this work are threefold: (1) it introduces the first NR index dedicated solely to spectral distortion in pansharpened images using MVG model, decoupling it from spatial quality assessment; (2) it integrates FDD and CM features within an MVG model, providing a robust statistical representation validated on diverse sensor data; and (3) extensive experiments on the NBU dataset demonstrate superior correlation with FR benchmarks (SAM, SID, CC) compared to QNR variants, highlighting its practical utility for algorithm optimization. This work provides targeted feature engineering and experimental protocols that allow future research and practitioners to extend and adapt the MVG framework to new satellites, sensors, or domain requirements.

The remainder of this paper is organized as follows. Section 2 details the proposed MVG-SDI methodology, including patching, feature extraction, model fitting, and score computation. Section 3 describes the experiments, including the datasets description, fusion algorithms, evaluation protocols, and analysis. Section 4 presents discussions, followed by conclusions in Section 5.

## 2. Proposed Method

This section details the No-Reference Multivariate Gaussian-based Spectral Distortion Index (MVG-SDI). The proposed approach operates on the hypothesis that spectral distortions in pansharpened images manifest as statistical deviations from the natural spectral characteristics inherent in the original MS data.

The MVG model is a statistical method that describes an image’s pixel distribution using a mean vector and a covariance matrix, which capture the average pixel values and the correlation between spectral bands, respectively. This model, first introduced for blind assessment of natural images by Mittal et al. [15], has proven effective at capturing the statistical regularities of natural scenes. In pansharpening assessment, the MVG model is fitted to both the original and the fused images. By comparing the statistical parameters of the two distributions, the model can quantify how well the pansharpened image preserves the original spectral information. For a *d*-dimensional feature vector f, the probability density function (PDF) of the MVG distribution is given by

(1) $$g\left(f\right)=\frac{1}{{\left(2\pi \right)}^{d/2}{\left|\Psi \right|}^{1/2}}\exp\left(-\frac{1}{2}{\left(f-\mu \right)}^{T}\Psi^{-1}\left(f-\mu \right)\right)$$

where: $f\in R^{d\times 1}$ is the feature vector, $\Psi \in R^{d\times d}$ is the covariance matrix, $\mu \in R^{d\times 1}$ is the mean vector, X denotes the feature matrix containing the image patches, *K* is the number of image patches, *d* is the dimensionality of the distribution, and the superscript *T* denotes the transpose.

**Figure 1:**
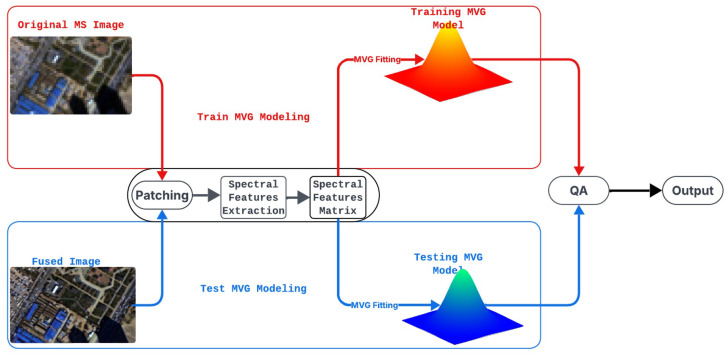
Flowchart of the proposed MVG-SDI method for spectral distortion assessment.

This work adapts the MVG framework specifically for spectral distortion by focusing on features sensitive to color and radiometric changes, while excluding spatial-oriented ones. As illustrated in Figure 1, the method compares the statistical characteristics of the fused image against an ideal reference derived from the original MS data. The process uses the MS image as the training reference and the fused image as the testing sample. Both undergo identical processing: patch division, spectral feature extraction (combining FDD from Benford’s Law and CMs), and aggregation into a Spectral Features Matrix. Separate MVG models are fitted into a training MVG model $\left(\mu_{\mathrm{ref}},\Psi_{\mathrm{ref}}\right)$ from the MS data, representing undistorted spectral properties, and a testing MVG model $\left(\mu_{\mathrm{test}},\Psi_{\mathrm{test}}\right)$ from the fused image. The Mahalanobis distance between these models quantifies distortion, with smaller values indicating better spectral preservation. This adaptation enhances robustness by accounting for feature covariances, enabling detection of subtle, inter-dependent spectral artifacts that simpler distances overlook.

### 2.1. Patch-Based Decomposition

The fused and MS images are decomposed into a uniform grid of non-overlapping 32×32 patches. Numerous patch sizes have been tested, and through empirical experimentation, this 32×32 size was determined to be the optimal choice. This patch-based approach directly addresses the spectral variability common in HR remote sensing imagery, where adjacent land-cover types such as vegetation, water, and urban surfaces display unique spectral characteristics. In contrast, a global statistical model would blend these diverse responses, potentially concealing specific distortions caused by pansharpening. The selected 32×32 dimension strikes a practical balance: it is expansive enough to ensure reliable statistical calculations for FDD and CMs, yet compact enough to maintain local uniformity. As a result, the MVG-SDI is capable of detecting fine, location-specific color variations or radiometric discrepancies that broader metrics would typically miss.

### 2.2. Spectral Feature Extraction

The performance of the proposed index depends on features that are highly sensitive to the spectral distortions typically introduced during the pansharpening process. To achieve this, a hybrid feature set was employed that combines FDD features derived from Benford’s Law to capture deviations in spectral statistics with CM features, which characterize the global color distribution through the mean, standard deviation, and skewness of each channel. This combination yields a comprehensive 21-dimensional feature vector for each image patch, effectively representing both local spectral consistency and global chromatic variation.

#### 2.2.1. First Digit Distribution Features

Distortion causes an image’s statistical characteristics to stray from their expected norms. By extracting these statistics as features and measuring their divergence, image quality can be assessed without any reference images. One widely used feature is Benford’s Law, which has been employed in natural image quality assessment [37,38]. Unlike natural images, remote sensing data often include multiple bands and rich spectral information, such as MS and hyperspectral images, making spectral distortion measurement in pansharpened MS images essential for evaluating sharpening performance.

To ensure the reproducibility of the proposed metric, it is important to note that no radiometric normalization, clipping, or dynamic range rescaling is applied to the input images prior to feature extraction. The HCS transform is applied directly to the raw pixel values of the fused and reference images. The only normalization performed is the scaling of the angular components $\theta_{k}$ by the constant 2/π, as defined in Equation (3) to map them to the [0,1] interval required for consistent First Digit Distribution analysis.

**Figure 2:**
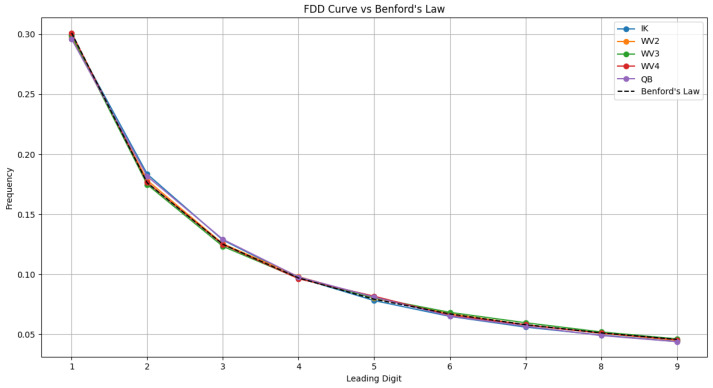
FDD features of pristine MS images from various sensors in NBU database.

The experiments on the NBU database show that the FDD of the angular components of pristine MS images in the HCD adheres to standard Benford’s Law. As shown in Figure 2, for high-quality (undistorted, unprocessed) images, the FDD features of four different MS bands align almost perfectly with the theoretical Benford distribution.

**Figure 3:**
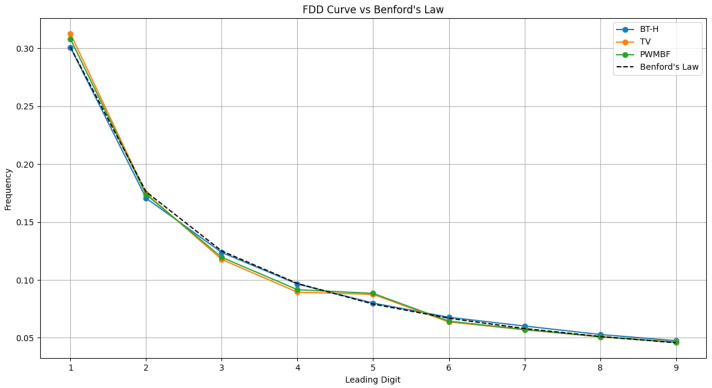
FDD of fused images from various sensors in NBU database.

**Figure 4:**
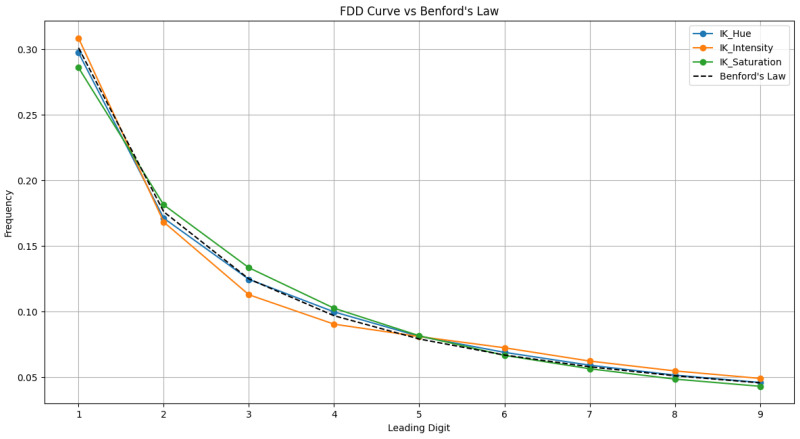
FDD features of distorted images from various sensors in NBU database.

Figure 3 illustrates that once the same images are processed by fusion algorithms known to introduce spectral errors (such as the BT-H, TV, and PWMBF methods), their first digit frequencies deviate markedly. Similarly, Figure 4 shows that simulated spectral degradations like hue, saturation, and non-linear intensity mismatch break the Benford pattern even more severely, causing the distribution to skew away from the theoretical curve. These distortions confirm that only pristine MS images in the HCD truly follow Benford’s Law; any filtering or spectrally altering processing disrupts the natural first digit statistics. Building on this, a nine-dimensional feature vector was extracted based on Benford’s Law to quantify spectral distortion.

First, the hyperspherical color space (HCS) transform is employed to map the *N*-band pansharpened image from its original space to the hyperspherical color space. This process separates the intensity component from the angular components, yielding one intensity component and N−1 angular components. Specifically, the intensity component characterizes the spatial information of the pansharpened image, while the angular components represent its spectral information.

Let the intensity component of the pansharpened image $\hat{M}=\left\{{\hat{M}}_{1},{\hat{M}}_{2},\ldots ,{\hat{M}}_{N}\right\}$ in the HCD be denoted as I, and the angular components be denoted as $\theta =\{\theta_{1},\theta_{2},\ldots ,\theta_{N-1}\}$. The dimensions of θ are H×W pixels, and the value range of each pixel in θ is [0,π/2]. The hyperspherical color transform is calculated as follows:

(2) $$\begin{array}{cc} I & =\sqrt{{\hat{M}}_{1}^{2}+{\hat{M}}_{2}^{2}+\cdots +{\hat{M}}_{N}^{2}}\\ \theta_{1} & =\tan^{-1}\left(\frac{\sqrt{{\hat{M}}_{2}^{2}+\cdots +{\hat{M}}_{N}^{2}}}{{\hat{M}}_{1}}\right)\\ \; & \vdots \\ \theta_{k} & =\tan^{-1}\left(\frac{\sqrt{{\hat{M}}_{k+1}^{2}+\cdots +{\hat{M}}_{N}^{2}}}{{\hat{M}}_{k}}\right)\\ \; & \vdots \\ \theta_{N-1} & =\tan^{-1}\left(\frac{{\hat{M}}_{N}}{{\hat{M}}_{N-1}}\right) \end{array}$$

The raw angular components $\theta_{k}$ fall within the range [0,π/2]. Before feature extraction, these are normalized to the range [0,1] to obtain ${\bar{\theta }}_{k}$:

(3) $${\bar{\theta }}_{k}=\frac{2}{\pi }\theta_{k}$$

**Figure 5:**
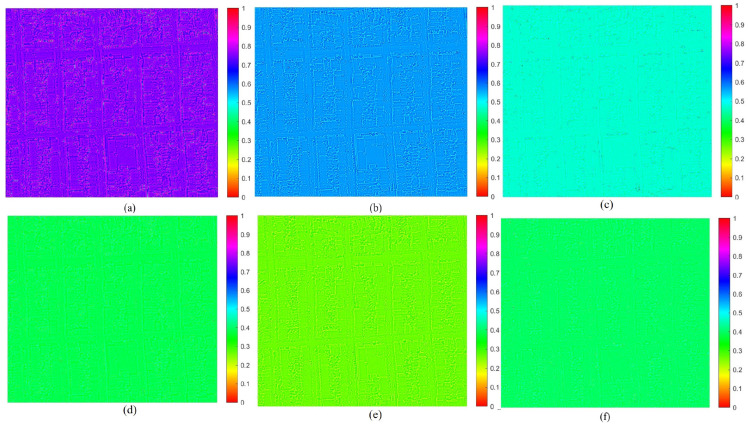
Heatmaps of HCD normalized angular components of the pansharpening results. (**a**) MS ${\bar{\theta }}_{1}$, (**b**) MS ${\bar{\theta }}_{2}$, (**c**) MS ${\bar{\theta }}_{3}$, (**d**) IHS ${\bar{\theta }}_{1}$, (**e**) IHS ${\bar{\theta }}_{2}$, and (**f**) IHS ${\bar{\theta }}_{3}$.

Figure 5 displays the heatmaps of the HCD normalized angular components for the MS image and the IHS pansharpened image, which exhibits severe spectral distortion. It can be observed that the normalized angular component heatmaps of the IHS pansharpened image differ significantly from those of the MS image. This demonstrates that the normalized angular components can effectively reflect spectral distortion.

Next, the FDD features are determined. For every pixel in the normalized component ${\bar{\theta }}_{k}$, the first non-zero digit is extracted. The probability of each digit a∈{1,2,…,9} is calculated as

(4) $$P_{k}\left(a\right)=\frac{Q_{a}}{H\times W}$$

where: $Q_{a}$ is the count of digit *a*, and H×W is the total number of pixels in the patch.

This creates a 9-dimensional FDD feature vector for each angle component. These are then averaged across the N−1 angles to produce a single 9-dimensional FDD feature vector $x_{FDD}$ for the patch:

(5) $$x_{FDD}=\frac{1}{N-1}\sum_{k=1}^{N-1}{\left[P_{k}\left(1\right)P_{k}\left(2\right),\ldots ,P_{k}\left(9\right)\right]}^{T}$$

where the superscript *T* represents the transpose of the vector.

#### 2.2.2. Color Moment Features

While the FDD features capture the underlying statistical distribution, they are complemented by CM features to provide a more direct and perceptually relevant measure of the image’s color profile. This feature set is designed to capture the global color distribution within each patch and is highly sensitive to the primary artifacts of spectral distortion, such as changes in brightness, chromaticity, and illumination. Pansharpening algorithms can often introduce radiometric shifts (biasing brightness), alter the gain between bands (changing color balance), or cause non-linear saturation, all of which are effectively quantified by this feature set.

For each patch, the first three statistical moments are calculated for each of the red (R), green (G), blue (B), and Near-Infrared (NIR) channels. This process forms a compact and highly descriptive 12-dimensional feature vector (4 channels × 3 moments/channel):

Mean (μ): The first-order moment. This represents the average color intensity of a channel, directly reflecting the image’s overall brightness or any radiometric bias introduced during fusion.
Standard deviation (σ): The second-order moment. This measures the contrast or dynamic range within a channel. A higher σ indicates more variation in pixel intensities, a property often compressed or unnaturally expanded by fusion.
Skewness (γ): The third-order moment. This captures the asymmetry of the pixel distribution. It is highly sensitive to non-linear distortions, indicating whether the channel is biased toward darker or brighter tones, which often results from pixel value clipping or saturation.

Thus, each image block yields the concatenated 12-dimensional feature vector:

(6) $$x_{CM}={\left[\mu_{R},\sigma_{R},\gamma_{R},\mu_{G},\sigma_{G},\gamma_{G},\mu_{B},\sigma_{B},\gamma_{B},\mu_{NIR},\sigma_{NIR},\gamma_{NIR}\right]}^{T}.$$

### 2.3. Spectral Feature Matrix Construction

Following the extraction of the two distinct feature sets from each 32×32 patch, the next step is to combine them into a single, powerful descriptor. For each individual image patch, the FDD features (a 9-dimensional vector) and the CM features (a 12-dimensional vector) are concatenated end-to-end. This fusion of features is crucial, as it creates a single, comprehensive vector that simultaneously describes the patch’s underlying statistical “naturalness” (from FDD) and its direct perceptual color profile (from CMs).

This process results in a final 21-dimensional spectral feature vector for each patch, defined as

(7) $$x=[x_{FDD},x_{CM}].$$

This procedure is repeated for all *N* non-overlapping patches extracted from the image. The resulting *N* feature vectors are then aggregated to form the comprehensive N×21 spectral feature matrix. This final matrix statistically represents the complete spectral properties of the entire image.

### 2.4. Model Fitting

The core of the quality assessment lies in comparing the statistical characteristics of the fused image against those of the original MS image using the MVG framework. To achieve this, two distinct models are constructed:

Training MVG model ($\mu_{ref},\Psi_{ref}$): This model is constructed from the spectral feature matrix (containing both FDD and CM features) extracted from the original, LR distortion-free MS image. Its parameters, the mean vector $\mu_{ref}$ and covariance matrix $\Psi_{ref}$, serve as the “ground truth” statistical benchmark, representing the ideal spectral properties that the fused image should replicate.
Testing MVG model ($\mu_{test},\Psi_{test}$): In parallel, a second model is built for the fused image under evaluation. Its mean vector $\mu_{test}$ and covariance matrix $\Psi_{test}$ are computed from its own spectral feature matrix, which also contains the FDD and Color Moment features. This model represents the actual spectral statistics of the final fused product, including any distortions.

The main steps of the proposed method can be summarized in Algorithm 1.
**Algorithm 1** Pseudocode of the proposed MVG-SDI method**Require:** Multispectral image ($I_{MS}$), fused image ($I_{Fused}$)**Require:** Block size S=32, feature dimension d=21**Ensure:** Spectral Distortion Index (*Q*)  1:**Step 1: Feature extraction**  2:*Function ExtractFeatures(Image):*  3:    Divide Image into *K* non-overlapping patches of size S×S  4:**for** k=1 to *K* **do**  5:    *// Extract FDD features (9 dimensions)*  6:    Convert patch to HCS to get angular components θ  7:    Normalize angles: $\bar{\theta }\leftarrow \theta \times \left(2/\pi \right)$  8:    Compute digit probabilities $x_{FDD}$ based on Benford’s Law  9:    *// Extract CM features (12 dimensions)*10:    Select first 4 bands (RGB + NIR)11:    Compute mean (μ), standard deviation (σ), and skewness (γ) for each band12:    Construct vector $x_{CM}=\left[\mu_{1},\sigma_{1},\gamma_{1},\ldots ,\mu_{4},\sigma_{4},\gamma_{4}\right]$13:    *// Concatenate feature vector*14:    $f_{k}\leftarrow \left[x_{FDD},x_{CM}\right]$15:**end for**16:**return** Feature matrix $F={\left[f_{1},f_{2},\ldots ,f_{K}\right]}^{T}$17:**Step 2: Model construction**18:$F_{ref}\leftarrow \mathrm{ExtractFeatures}\left(I_{MS}\right)$19:$F_{test}\leftarrow \mathrm{ExtractFeatures}\left(I_{Fused}\right)$20:Compute training MVG model parameters:21:$\mu_{ref}\leftarrow \mathrm{mean}\left(F_{ref}\right)$, $\Psi_{ref}\leftarrow \mathrm{cov}\left(F_{ref}\right)$22:Compute testing MVG model parameters:23:$\mu_{test}\leftarrow \mathrm{mean}\left(F_{test}\right)$, $\Psi_{test}\leftarrow \mathrm{cov}\left(F_{test}\right)$24:**Step 3: Distance calculation**25:Compute pooled covariance: $\Psi \leftarrow (\Psi_{ref}+\Psi_{test})/2$26:Calculate difference vector: $\Delta \mu \leftarrow \mu_{ref}-\mu_{test}$27:Compute Mahalanobis distance:28:$Q\leftarrow \sqrt{\Delta \mu {\left(\Psi \right)}^{-1}\Delta \mu^{T}}$29:**return** *Q*

### 2.5. Quality Score Computation

The spectral distortion is formally quantified by calculating the Mahalanobis distance (*D*) between the statistical parameters of the two fitted MVG models, utilizing the formulation provided in Equation (8). This distance measures the dissimilarity between the mean feature vector of the test model ($\mu_{test}$) and the mean of the ideal reference model ($\mu_{ref}$).

By incorporating the pooled covariance matrix, this metric offers a more robust assessment than simple Euclidean distance. It explicitly normalizes for statistical variance and accounts for the complex inter-dependencies between distinct spectral features, such as the correlation between FDD variations and Color Moments.

(8) $$D=\sqrt{{\left(\mu_{ref}-\mu_{test}\right)}^{T}\Psi^{-1}\left(\mu_{ref}-\mu_{test}\right)}$$

## 3. Experiments

### 3.1. Datasets

Experimental validation was performed using the publicly available large-scale NBU dataset [34], which comprises 1200 diverse image pairs acquired by five distinct satellite sensors: IK, WV-2, WV-3, and WV-4. Each sample pair contains a high-resolution PAN image (1024×1024 pixels) and a corresponding low-resolution MS image (256×256 pixels).

**Figure 6:**
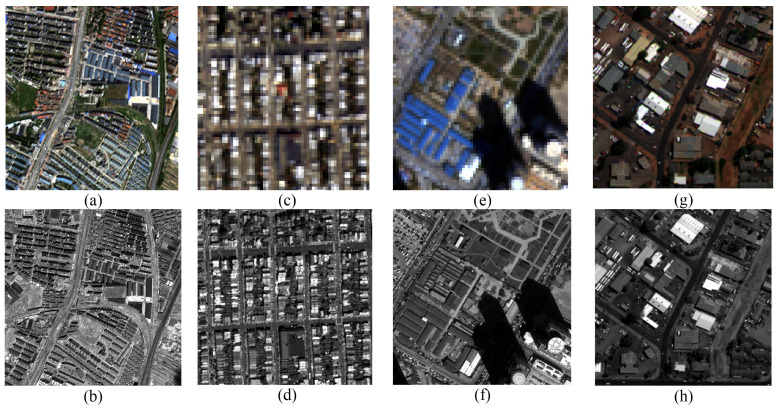
MS and PAN image samples from IK (**a**,**b**), WV-2 (**c**,**d**), WV-3 (**e**,**f**), and WV-4 (**g**,**h**).

Table 1 provides a detailed breakdown of the image pairs and spectral bands, while Figure 6 displays representative examples from each sensor subset.

### 3.2. Fusion Algorithms

The evaluation employed 19 distinct pansharpening algorithms, sourced from two public MATLAB toolkits [39] to ensure standardized implementation. These methods were selected to represent a diverse cross-section of established techniques, which is crucial for assessing the proposed index’s performance across various types of spectral artifacts. The set includes five CS, nine MRA, four VO, and four ML methods, covering the most prominent categories in the field. Table 2 provides a summary of the algorithms utilized in this study.

### 3.3. Implementation Details

To ensure the reliability of the proposed MVG-SDI metric and facilitate future comparisons, all key implementation parameters have been standardized. These settings, including patch size, normalization constants, and feature dimensionality. The specific values used in this study are detailed in Table 3 below.

### 3.4. Spectral Quality Assessment Metrics

To validate the performance of the proposed MVG-SDI, its results were compared against a comprehensive suite of established evaluation metrics. This suite included both competing NR methods and benchmark FR measures.

For the NR comparison, the spectral distortion components of several prominent QNR-like indices, namely ${\mathrm{QNR}}_{\lambda }$, ${\mathrm{FQNR}}_{\lambda }$, and ${\mathrm{MQNR}}_{\lambda }$, were employed. These indices are designed to assess how well spectral characteristics are preserved in pansharpened images without requiring a ground truth reference.

In addition, three widely used FR evaluation measures, the Spectral Angle Mapper (SAM), Spectral Information Divergence (SID), and the correlation coefficient (CC), were incorporated as benchmarks. These metrics are employed specifically to quantify spectral angular consistency, Spectral Information Divergence, and statistical correlation, respectively.

Moreover to properly evaluate these metrics, this study employs a dual-scale experimental protocol consisting of NR and FR validation. This approach is essential for comparing the proposed index against other NR metrics at the original scale, while validating its performance against FR benchmarks at a reduced scale. The two protocols are summarized as follows.

#### 3.4.1. No-Reference Assessment

This protocol compares the proposed spectral index against existing NR spectral indexes (${\mathrm{QNR}}_{\lambda }$, ${\mathrm{FQNR}}_{\lambda }$, and ${\mathrm{MQNR}}_{\lambda }$). The fusion algorithms are applied at the original full resolution (producing 1024×1024 fused images), and all NR metrics are computed directly on these outputs to evaluate performance under realistic conditions.

#### 3.4.2. Reduced-Resolution Validation (Wald’s Protocol)

This protocol validates the proposed NR index against reference-based scores using Wald’s protocol. Following standard degradation and upsampling procedures, fusion is performed at a reduced scale (256×256 images). The FR metrics (SAM, SID, and CC) are computed by comparing the fused results against the original 256×256 MS image, which serves as the ground truth. The proposed NR spectral index is simultaneously calculated on these reduced-resolution images to analyze its correlation with the FR benchmarks.

### 3.5. Numerical Evaluation

A comprehensive analysis was conducted to benchmark the proposed MVG-SDI against state-of-the-art metrics across diverse fusion categories (CS, MRA, VO, and ML). In the reported results, the optimal performance within each category is highlighted in red, while the poorest performance is marked in blue.

A critical observation from this comparison is the strong alignment between the proposed method and the advanced ${\mathrm{FQNR}}_{\lambda }$ model. As evidenced in Table 4, Table 5, Table 6 and Table 7, the proposed index exhibits a high degree of correlation with ${\mathrm{FQNR}}_{\lambda }$ in identifying extreme performers. Specifically, both metrics converge on the same best-performing algorithm in the WV-2 dataset, identify identical best and worst algorithms in the WV-3 dataset, and consistently flag the same poorest performer in the WV-4 dataset.

Furthermore, the numerical results validate the proposed method’s capability to assess spectral distortion effectively. It demonstrates superior performance to ${\mathrm{MQNR}}_{\lambda }$ and comparable to established NR benchmarks, while maintaining a logical consistency with FR metrics such as the CC.

The numerical results for the IK dataset are presented in Table 4. PNN achieved the best scores for CC, SAM, and SID, indicating superior spectral fidelity with respect to the reference. Conversely, GS yielded the lowest CC value, while PWMBF recorded the poorest performance for both SAM and SID. Among the QNR-based indices, optimal selection varied: ${\mathrm{QNR}}_{\lambda }$ favored A-PNN, ${\mathrm{FQNR}}_{\lambda }$ selected SR-D, and ${\mathrm{MQNR}}_{\lambda }$ identified GS as the top-performing algorithm. Notably, the proposed method identified PRACS as the best fusion model, aligning more closely with the preferences of ${\mathrm{MQNR}}_{\lambda }$ for CS-based methods. Regarding the poorest results, SR-D exhibited the highest distortion levels according to the proposed metric, whereas ${\mathrm{MQNR}}_{\lambda }$ flagged MTF-GLP-HPM-H as the worst performer.

Quantitative results for the WV-2 dataset are presented in Table 5. PNN-IDX achieved the best scores for both SAM and SID, indicating superior spectral fidelity, while MTF-GLP-HPM-H recorded the highest CC value. Conversely, MF yielded the lowest correlation coefficient, while PRACS and AWLP exhibited the highest spectral distortion across both SAM and SID measures. Among the QNR-based indices, optimal selection varied: ${\mathrm{QNR}}_{\lambda }$ favored BT-H, whereas both ${\mathrm{MQNR}}_{\lambda }$ and ${\mathrm{FQNR}}_{\lambda }$ identified GS as the top-performing algorithm. Notably, the proposed method aligned with these latter indices, consistently identifying GS as the best fusion model. Regarding the poorest results, the proposed metric flagged SR-D as the worst performer, while ${\mathrm{MQNR}}_{\lambda }$ identified PNN-IDX, the algorithm with the best ground truth spectral fidelity, as the poorest model, further highlighting the divergence between FR and NR assessments.

The numerical assessment results for the WV-3 dataset are summarized in Table 6. MTF-GLP achieved the highest CC, while BT-H and A-PNN-FT recorded the best performances for SID and SAM, respectively, indicating superior spectral preservation. Conversely, PNN-IDX consistently yielded the poorest results across all full-reference metrics, exhibiting the lowest correlation and highest spectral distortion. Among the QNR-based indices, optimal selection varied significantly: ${\mathrm{QNR}}_{\lambda }$ and ${\mathrm{MQNR}}_{\lambda }$ favored BT-H, aligning with the SID results, whereas ${\mathrm{FQNR}}_{\lambda }$ selected GS as the top-performing algorithm. Notably, the proposed method also identified GS as the best fusion model. Regarding the poorest results, a distinct contradiction was observed: the proposed metric flagged BT-H as the worst performer (0.2899) despite it achieving the best SID score, while ${\mathrm{MQNR}}_{\lambda }$ aligned with the FR benchmarks by identifying PNN-IDX as the poorest model.

The quantitative evaluation for the WV-4 dataset is detailed in Table 7. FE-HPM achieved the best scores for CC, SAM, and SID, consistently demonstrating superior spectral fidelity across all full-reference benchmarks. Conversely, BT-H recorded the lowest correlation coefficient, while SR-D and PWMBF exhibited the highest spectral distortion in terms of SID and SAM, respectively. Among the QNR-based indices, the optimal selection varied: ${\mathrm{QNR}}_{\lambda }$ favored BDSD, ${\mathrm{MQNR}}_{\lambda }$ selected PRACS, and ${\mathrm{FQNR}}_{\lambda }$ identified SR-D as the top-performing algorithm. Notably, the proposed method identified PWMBF as the best fusion model; however, this presents a significant contradiction, as PWMBF yielded the poorest SAM value in the reference-based assessment. Regarding the poorest results, a rare consensus was observed: all NR metrics, including the proposed method, flagged BT-H as the worst performer, aligning with its lowest ranking in the CC evaluation.

### 3.6. Visual Evaluation

The visual performance of the proposed index was evaluated against the benchmark metrics (${\mathrm{QNR}}_{D_{\lambda }}$, ${\mathrm{FQNR}}_{D_{\lambda }}$, and ${\mathrm{MQNR}}_{D_{\lambda }}$) using the GS fusion method, which is selected for its tendency to induce noticeable spectral distortions.

**Figure 7:**
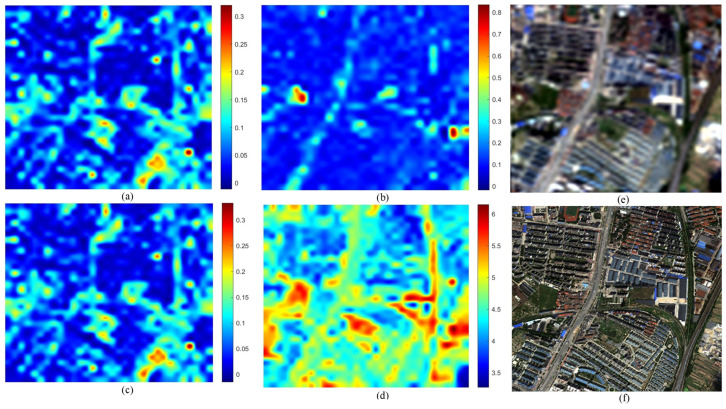
Visual comparison on the IK dataset: (**a**) ${\mathrm{QNR}}_{D_{\lambda }}$, (**b**) ${\mathrm{FQNR}}_{D_{\lambda }}$, (**c**) ${\mathrm{MQNR}}_{D_{\lambda }}$, (**d**) proposed, (**e**) MS, and (**f**) GS.

Figure 7 illustrates the distortion maps for the IK dataset. The GS fused image (f) exhibits characteristic spectral shifts relative to the MS reference (e). The maps for ${\mathrm{QNR}}_{D_{\lambda }}$ (a) and ${\mathrm{MQNR}}_{D_{\lambda }}$ (c) display identical patterns concentrated almost exclusively along high-frequency edges. This indicates a bias toward spatial features rather than true spectral deviations. Meanwhile, ${\mathrm{FQNR}}_{D_{\lambda }}$ (b) appears almost entirely blue, failing to register the distortion. Conversely, the proposed index (d) generates a distinct heatmap with high-intensity values (red and yellow) distributed across broad object surfaces, effectively highlighting the spectral errors that competing metrics mistake for spatial structures.

**Figure 8:**
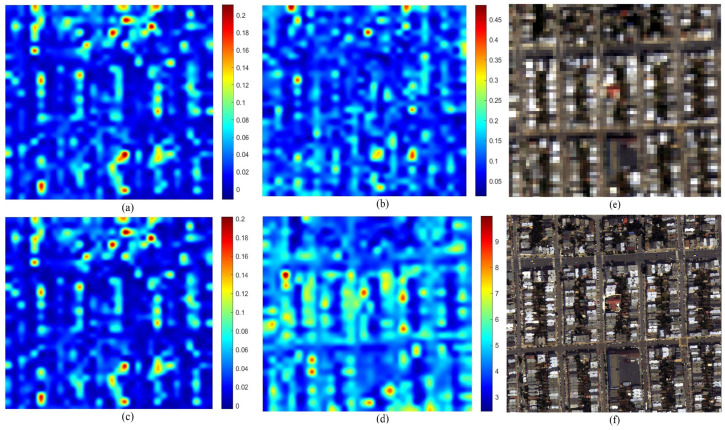
Visual comparison on the WV-2 dataset: (**a**) ${\mathrm{QNR}}_{D_{\lambda }}$, (**b**) ${\mathrm{FQNR}}_{D_{\lambda }}$, (**c**) ${\mathrm{MQNR}}_{D_{\lambda }}$, (**d**) proposed, (**e**) MS, and (**f**) GS.

This behavior is further validated on the WV-2 dataset, as shown in Figure 8. Here, the GS method introduces spectral deviations across varied urban materials. Consistent with the previous dataset, ${\mathrm{QNR}}_{D_{\lambda }}$ (a) and ${\mathrm{MQNR}}_{D_{\lambda }}$ (c) remain nearly indistinguishable, focusing narrowly on high-contrast features such as bright rooftops, while ${\mathrm{FQNR}}_{D_{\lambda }}$ (b) significantly underestimates the error magnitude. The proposed index (d), however, captures the widely distributed inconsistencies, producing a heatmap that correlates robustly with the global spectral degradation inherent to the component substitution process.

**Figure 9:**
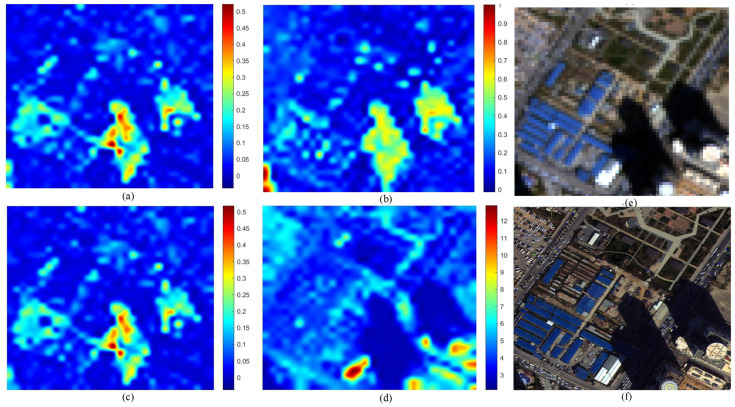
Visual comparison on the WV-3 dataset: (**a**) ${\mathrm{QNR}}_{D_{\lambda }}$, (**b**) ${\mathrm{FQNR}}_{D_{\lambda }}$, (**c**) ${\mathrm{MQNR}}_{D_{\lambda }}$, (**d**) proposed, (**e**) MS, and (**f**) GS.

The analysis of the WV-3 dataset shown in Figure 9 demonstrates the robustness of the proposed index in scenes with high dynamic range. Visually, the GS image (f) shows spectral shifts in both bright blue industrial rooftops and deep shadowed regions. The competing metrics exhibit a strong radiance bias: ${\mathrm{QNR}}_{D_{\lambda }}$ and ${\mathrm{MQNR}}_{D_{\lambda }}$ detect artifacts only on the bright rooftops, leaving the background and shadows unassessed. In contrast, the proposed index (d) identifies spectral degradation across the entire dynamic range, showing significant responsiveness even in the low-radiance shadowed areas that other metrics fail to register.

**Figure 10:**
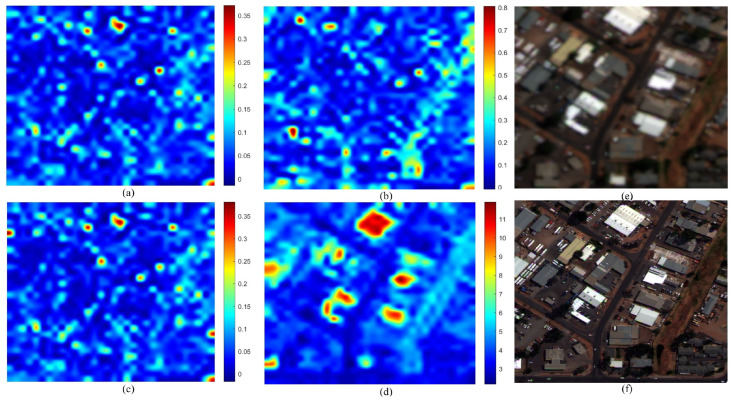
Visual comparison on the WV-4 dataset: (**a**) ${\mathrm{QNR}}_{D_{\lambda }}$, (**b**) ${\mathrm{FQNR}}_{D_{\lambda }}$, (**c**) ${\mathrm{MQNR}}_{D_{\lambda }}$, (**d**) proposed, (**e**) MS, and (**f**) GS.

Finally, the WV-4 dataset, as shown in Figure 10, offers the most striking validation. The GS fusion (f) suffers from severe global spectral distortion, appearing as an unnatural reddish-brown hue shift compared to the reference (e). Despite this obvious degradation, ${\mathrm{FQNR}}_{D_{\lambda }}$ (b) remains unresponsive, and ${\mathrm{QNR}}_{D_{\lambda }}$ (a) and ${\mathrm{MQNR}}_{D_{\lambda }}$ (c) display only scattered, low-intensity noise. The proposed index (d) is the only metric to produce a high-intensity response with prominent hotspots aligning precisely with the most distorted regions, proving its superior capability in quantifying severe spectral artifacts.

### 3.7. Visual Analysis of Fusion Results

**Figure 11:**
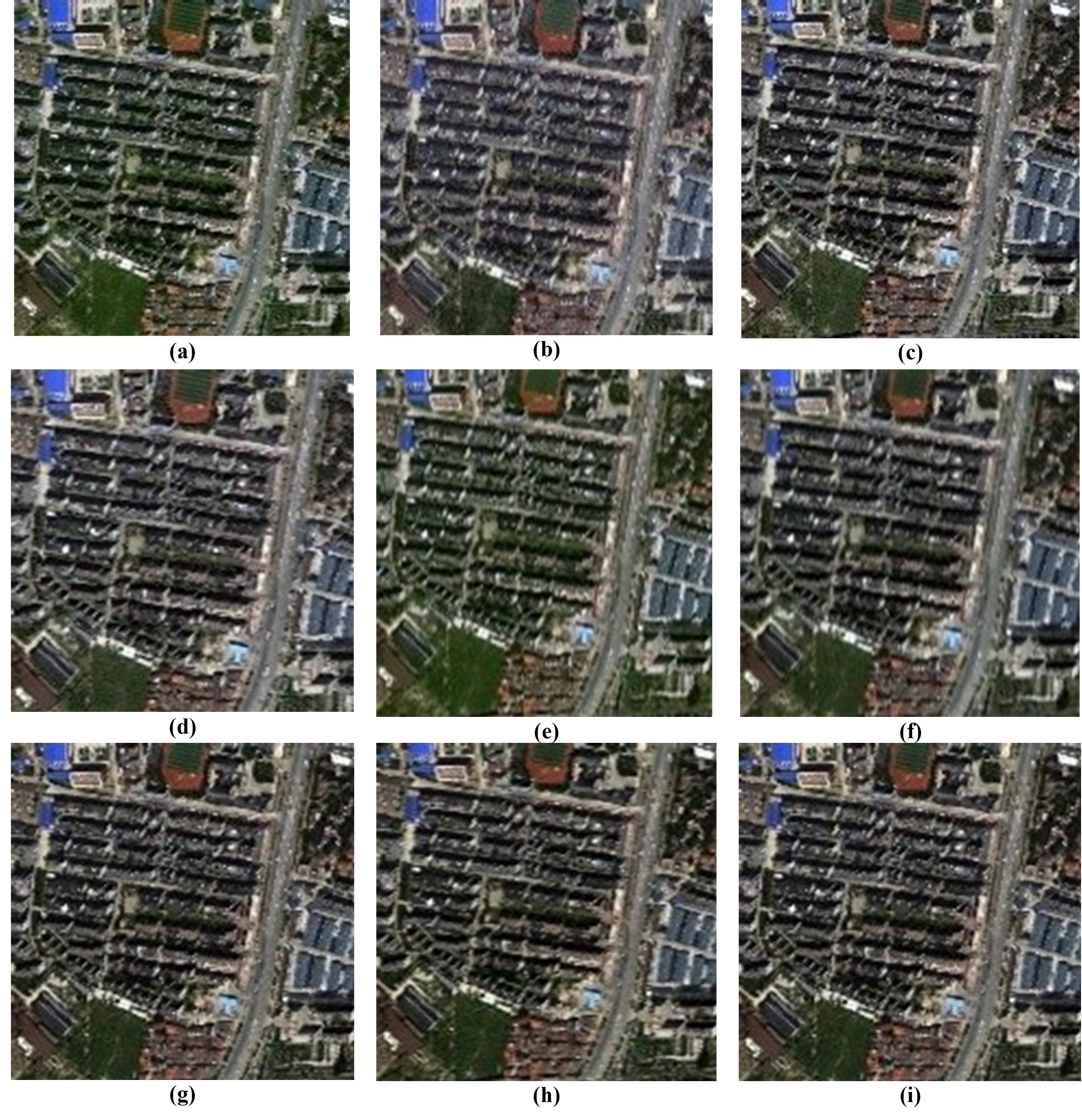
Close-ups of the main fusion results for the IK dataset (bands: RGB): (**a**) BT-H, (**b**) BDSD, (**c**) GS, (**d**) AWLP, (**e**) MTF-GLP, (**f**) MTF-GLP-HPM-FS, (**g**) PNN, (**h**) A-PNN, and (**i**) A-PNN-FT.

**Figure 12:**
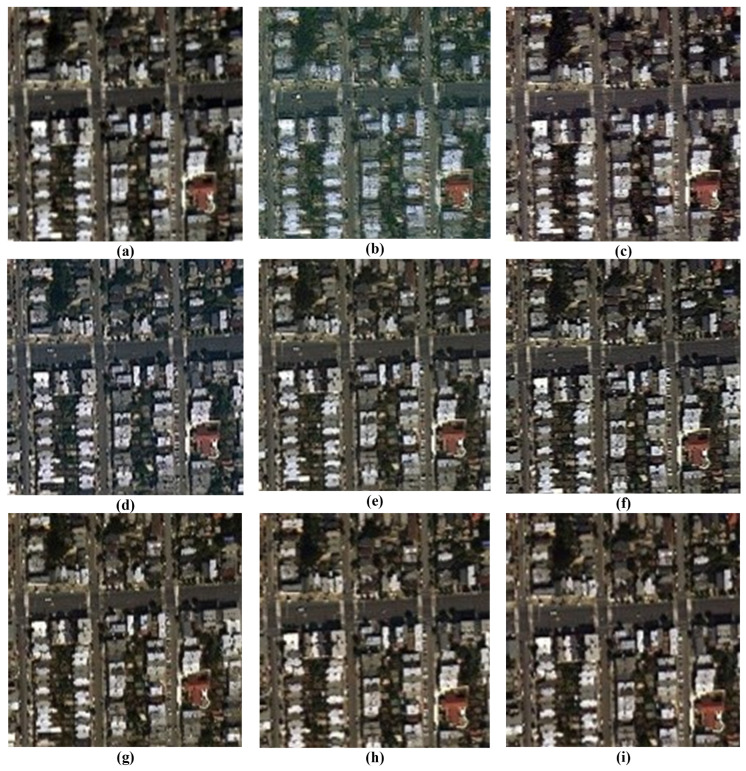
Close-ups of the main fusion results for the WV-2 dataset (bands: RGB): (**a**) BT-H, (**b**) BDSD, (**c**) GS, (**d**) AWLP, (**e**) MTF-GLP, (**f**) MTF-GLP-HPM-FS, (**g**) PNN, (**h**) A-PNN, and (**i**) A-PNN-FT.

**Figure 13:**
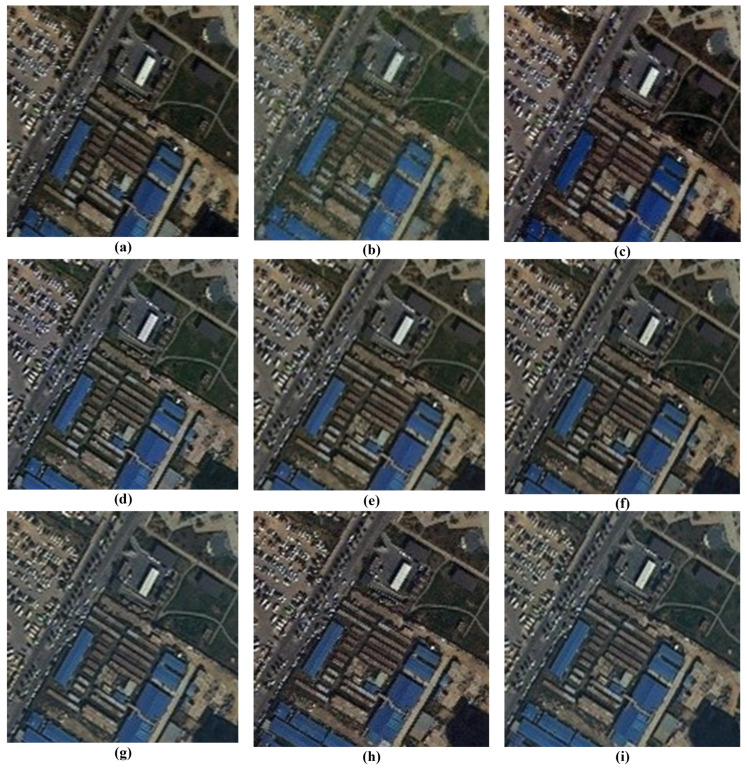
Close-ups of the main fusion results for the WV-3 dataset (bands: RGB): (**a**) BT-H, (**b**) BDSD, (**c**) GS, (**d**) AWLP, (**e**) MTF-GLP, (**f**) MTF-GLP-HPM-FS, (**g**) PNN, (**h**) A-PNN, and (**i**) A-PNN-FT.

**Figure 14:**
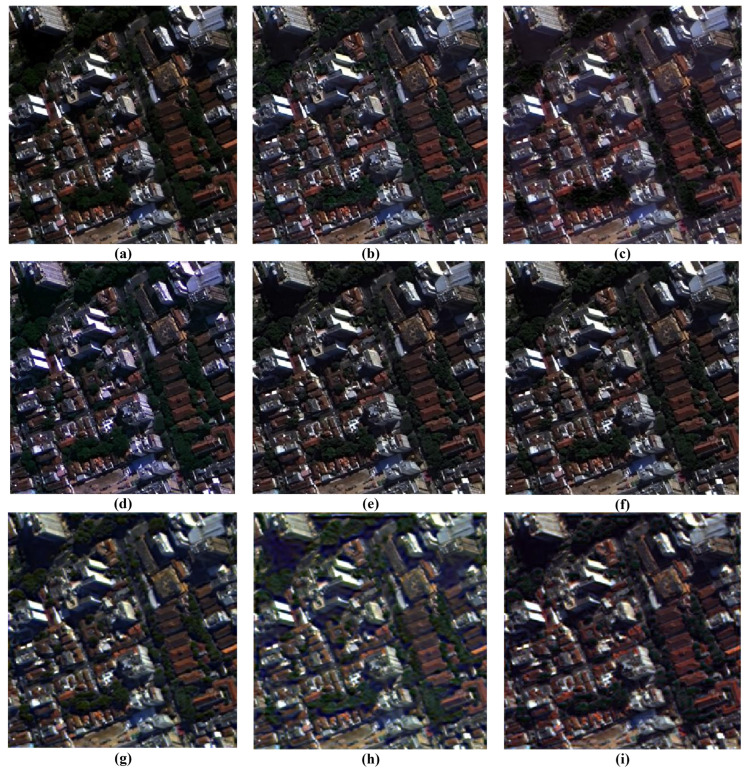
Close-ups of the main fusion results for the WV-4 dataset (bands: RGB): (**a**) BT-H, (**b**) BDSD, (**c**) GS, (**d**) AWLP, (**e**) MTF-GLP, (**f**) MTF-GLP-HPM-FS, (**g**) PNN, (**h**) A-PNN, and (**i**) A-PNN-FT.

A comprehensive visual inspection of the fusion outcomes across the different datasets, ranging from Figure 11, Figure 12, Figure 13 and Figure 14, reveals distinct variations in algorithmic performance. This qualitative analysis highlights the critical trade-off between spatial enhancement and spectral preservation, demonstrating how certain methods generalize more robustly across diverse sensor platforms than others.

For the IK dataset illustrated in Figure 11, the BT-H and MTF-GLP methods distinguish themselves with superior visual performance. These algorithms effectively inject high-frequency spatial details while maintaining rigorous spectral fidelity, resulting in images that are sharp yet natural. The PNN method also delivers competent results, striking a commendable balance between detail enhancement and artifact suppression. In contrast, the BDSD algorithm performs with mediocrity, failing to achieve the spatial crispness defined by the top performers. Meanwhile, the GS and AWLP methods occupy a middle ground; while their outputs are acceptable, they lack the refined clarity and spectral accuracy observed in the leading models.

In the case of the WV-2 dataset presented in Figure 12, a sharp disparity in spectral preservation capabilities is evident. The BT-H and GS algorithms, alongside the deep learning-based family (PNN, PNN-IDX, and A-PNN), produce the most visually convincing results. These methods are characterized by the precise rendering of spatial features and the maintenance of natural color distributions. Conversely, both BDSD and AWLP suffer from severe spectral degradation that compromises image utility. Specifically, BDSD introduces a pervasive, unnatural green hue, whereas AWLP manifests a distinct brownish cast, indicating a significant failure to preserve the original spectral distribution of the scene.

The visual assessment of the WV-3 dataset in Figure 13 highlights the robustness of BT-H, GS, and PNN-IDX, which consistently provide very good visual results with high spatial clarity. AWLP also performs well in this scenario. However, spectral distortions remain a challenge for other methods: BDSD again exhibits a strong green bias, while PNN produces an unclear, brownish output. Furthermore, A-PNN fails to retain visual quality, resulting in a generally poor fusion product.

Finally, for the WV-4 dataset presented in Figure 14, the traditional methods largely outperform the learning-based approaches. BT-H, BDSD, GS, MTF-GLP, and MTF-GLP-HPM-FS all demonstrate good fusion capabilities, balancing spatial enhancement with spectral accuracy. AWLP, however, results in noticeably darker imagery, suggesting a loss of luminance. Notably, the deep learning models (PNN, PNN-IDX, and A-PNN) struggle significantly with this dataset, collectively exhibiting severe spectral shifts towards yellow and green tones, rendering them less suitable for this specific sensor data.

### 3.8. Quality Assessment with Spectral Degradations

Different pansharpening methods can introduce various color-related artifacts, known as spectral degradations. Common spectral degradations such as color shifting, intensity mismatch, and oversaturation significantly reduce the spectral fidelity of the fused image. To test the proposed method, these spectral degradations were manually generated to verify its efficacy in correct identification and ranking.

#### 3.8.1. Hue and Saturation Shift

These artifacts represent one of the most direct and perceptually obvious sources of spectral distortion. They are particularly common in CS methods, most notably the IHS fusion family. The core issue arises from a fundamental spectral mismatch between the two source images. In the IHS method, the HR MS image is transformed into the IHS color space, and its “intensity” (I) component is replaced by the high-resolution PAN image. The problem is that the broad spectral response of the PAN sensor is not a perfect representation of the synthetic intensity component, which is calculated from the narrower MS bands. When this spectrally inconsistent PAN image is substituted and transformed back to the original color space, it introduces brightness levels that do not align with the original hue (H) and saturation (S) information. This mismatch leads to significant and unrealistic color shifts, altering the appearance of features like vegetation or water [5].

To systematically simulate and test the proposed index’s sensitivity to this degradation, the image is converted to the HSV (or HSI) color space, where the chromatic (H, S) and brightness (V) components can be manipulated independently. A hue shift is simulated by adding a constant offset, ΔH, to the entire hue channel. This effectively “rotates” all colors on the color wheel, creating a global color cast (e.g., shifting all blues toward purple).

(9) $$H_{\mathrm{distorted}}=\left(H_{\mathrm{original}}+\Delta H\right)\left(\mathrm{mod}\;1\right)$$

A saturation shift is simulated by multiplying the saturation channel by a scaling factor, $\alpha_{S}$. This modifies the “purity” or “vividness” of the colors.

(10) $$S_{\mathrm{distorted}}=S_{\mathrm{original}}\times \alpha_{S}$$

The severity of the distortion is precisely controlled by the magnitude of ΔH (a larger shift) and how far the scaling factor $\alpha_{S}$ deviates from one (with $\alpha_{S}>1$ causing oversaturation and $\alpha_{S}<1$ causing desaturation or “washout”).

#### 3.8.2. Non-Linear Intensity Mismatch

This artifact represents a complex form of spectral distortion where the brightness of the image is altered in a non-linear fashion. Unlike a simple, uniform brightening or darkening, this mismatch disproportionately affects different intensity levels, often compressing or expanding the mid-tones while leaving the darkest and brightest pixels relatively unchanged. This frequently leads to a “washed-out” or, conversely, an “overly dark” and “crushed” appearance.

This type of distortion is a common failure mode in MRA methods. These algorithms work by extracting high-frequency spatial details from the PAN image and then using an “injection model” to add them to the up-sampled MS bands. The amount of detail added is controlled by injection gains. When these gains are incorrectly calculated, either by “over-injecting” (adding too much detail) or “under-injecting” (adding too little), the resulting change in brightness is not uniform. Such non-linear intensity shifts can significantly alter the image’s radiometric values, which is particularly detrimental for quantitative analysis as it corrupts the accuracy of derived products like vegetation indices [6]. This non-linear degradation is effectively simulated by applying a power function, commonly known as gamma correction, to the image. To isolate the brightness component from the color information, this operation is performed on the value (V) channel in the HSV color space:

(11) $$V_{\mathrm{distorted}}={\left(V_{\mathrm{original}}\right)}^{\gamma }$$

The severity of this mismatch is controlled by the gamma parameter (γ). A γ value of 1.0 results in no change. However, a value of γ<1 brightens the image by boosting the mid-tones, simulating the washed-out effect of over-injection. Conversely, a value of γ>1 darkens the image by compressing the mid-tones, mimicking the effect of under-injection. The further γ deviates from 1.0, the more severe the non-linear spectral distortion.

### 3.9. Experimental Results with Spectral Degradations

#### 3.9.1. Objective Evaluation

This experiment evaluates the robustness of the proposed metric against manually induced spectral degradations. The primary objective is to verify the monotonicity of the metric; that is, the quality score should exhibit a consistent increase as the severity of the spectral distortion (specifically the hue shift, saturation shift, and non-linear intensity gamma) increases. A higher score must reliably indicate a strictly worse result without suffering from premature saturation or insensitivity.

**Figure 15:**
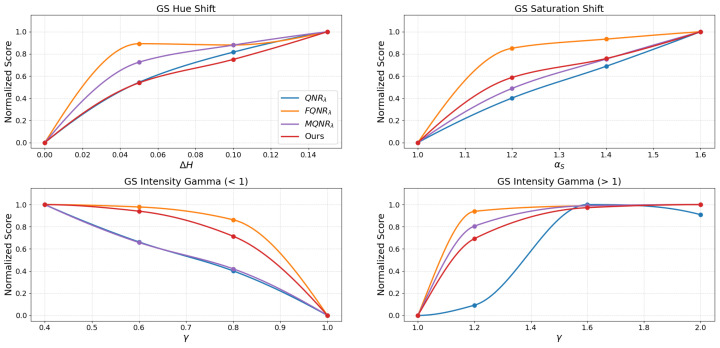
The evaluation of the degradations on the IK dataset using the GS method.

**Figure 16:**
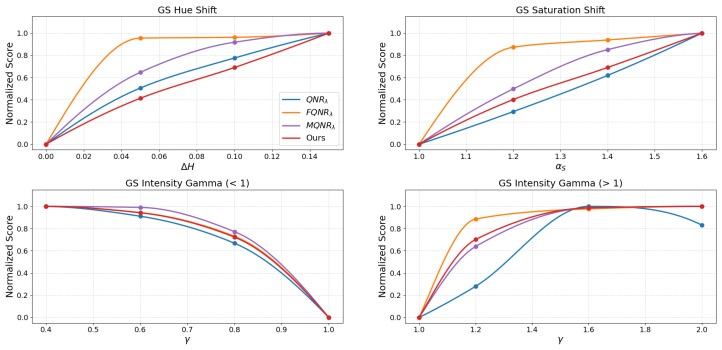
The evaluation of the degradations on the WV-2 dataset using the GS method.

**Figure 17:**
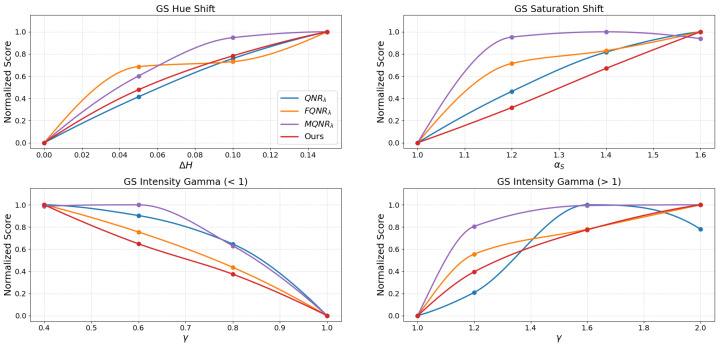
The evaluation of the degradations on the WV-3 dataset using the GS method.

**Figure 18:**
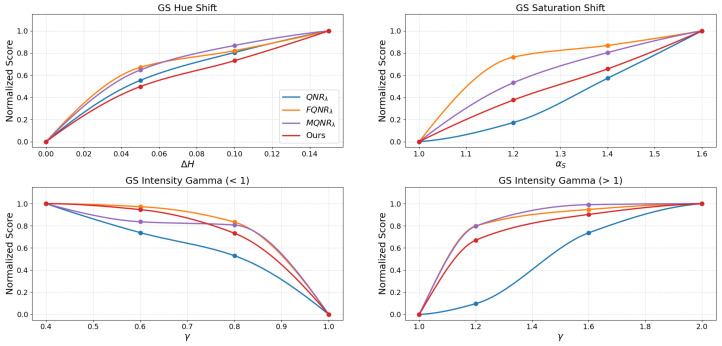
The evaluation of the degradations on the WV-4 dataset using the GS method.

The assessment of the continuous trends presented in Figure 15, Figure 16, Figure 17 and Figure 18 benchmarks the performance of the proposed method against established indices: ${\mathrm{QNR}}_{\lambda }$, ${\mathrm{MQNR}}_{\lambda }$, and ${\mathrm{FQNR}}_{\lambda }$.

For the GS method on the IK dataset, as shown in Figure 15, the proposed method demonstrates superior capability in quantifying spectral distortions.

In the saturation shift analysis, the ${\mathrm{FQNR}}_{\lambda }$ curve exhibits excessive sensitivity, marked by a steep initial spike to a near-maximum score (>0.8) at the lowest degradation level ($\alpha_{S}=1.20$), indicating premature saturation. In contrast, the proposed method follows a nearly linear trajectory with a consistent, gradual rise in penalty. Furthermore, in the intensity gamma evaluation (γ>1), standard ${\mathrm{QNR}}_{\lambda }$ displays a significant lack of sensitivity, remaining flat, whereas ${\mathrm{FQNR}}_{\lambda }$ saturates rapidly. The proposed method avoids these extremes, yielding a balanced and monotonic curve that accurately reflects the increasing magnitude of degradation.

An assessment of the WV-2 dataset, as shown in Figure 16, reinforces these findings. The ${\mathrm{FQNR}}_{\lambda }$ curve again exhibits excessive sensitivity to saturation shifts, spiking early, while the proposed method maintains a consistent, gradual trajectory. Similarly, in the intensity gamma evaluation (γ>1), standard ${\mathrm{QNR}}_{\lambda }$ remains insensitive (score <0.2 at γ=1.20), while ${\mathrm{FQNR}}_{\lambda }$ rises sharply. The proposed method provides a stable compromise, offering a balanced and monotonic response.

The results for the WV-3 dataset, shown in Figure 17, corroborate the robust stability of the proposed index, particularly where others fail. Here, ${\mathrm{MQNR}}_{\lambda }$ exhibits a critical failure in the saturation shift test, plateauing immediately (>0.9) at the initial degradation level ($\alpha_{S}=1.20$).

Conversely, the proposed method initiates at a moderate penalty (∼0.3) and rises monotonically. A similar trend is observed for the intensity gamma, where the proposed metric bridges the gap between the insensitivity of ${\mathrm{QNR}}_{\lambda }$ and the premature saturation of ${\mathrm{MQNR}}_{\lambda }$, ensuring a reliable assessment of radiometric consistency.

Finally, an evaluation on the WV-4 dataset, as shown in Figure 18, demonstrates the proposed method’s balanced sensitivity. While ${\mathrm{FQNR}}_{\lambda }$ reacts disproportionately to initial saturation shifts (score >0.75) and ${\mathrm{QNR}}_{\lambda }$ remains largely unresponsive, the proposed method exhibits a moderate, monotonic trajectory (reaching ∼0.38 at $\alpha_{S}=1.20$). In intensity gamma tests, it avoids the negligible response of ${\mathrm{QNR}}_{\lambda }$ and the sharp spikes of ${\mathrm{FQNR}}_{\lambda }$ and ${\mathrm{MQNR}}_{\lambda }$, providing a strictly monotonic response curve that effectively characterizes varying degrees of spectral degradation.

#### 3.9.2. Visual Analysis with Spectral Degradations

**Figure 19:**
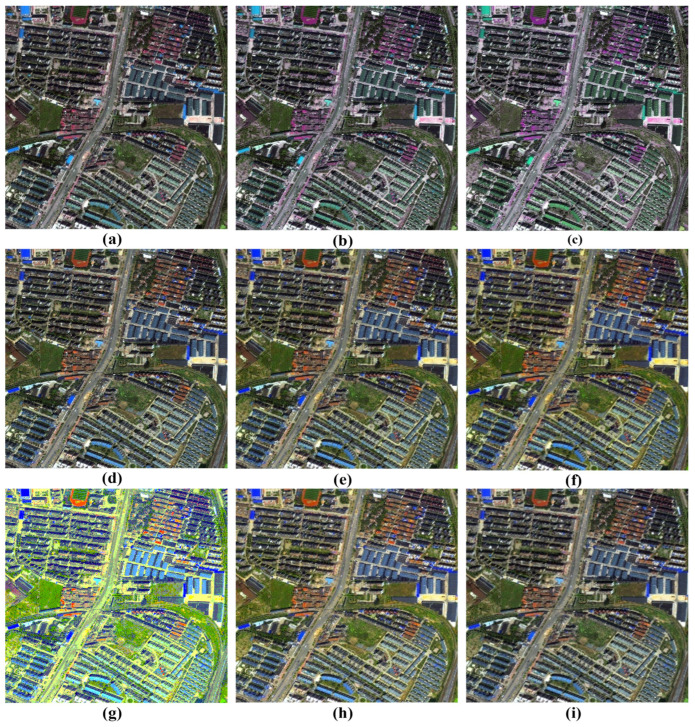
Visual examples of simulated spectral degradations. The top row (**a**–**c**) shows hue shift artifacts with ΔH values of 0.05, 0.10, and 0.15. The middle row (**d**–**f**) shows saturation shift applied with a factor $\alpha_{S}$ of 1.2, 1.4, and 1.6. The bottom row (**g**–**i**) shows intensity gamma correction applied with a γ value of 0.4, 0.6, and 0.8.

Figure 19 provides a visual analysis of a GS-fused image from the IK dataset, showing three types of manually generated spectral distortions at different severity levels. The figure is organized in a 3×3 grid, with each row dedicated to one type of artifact.

The top row (a, b, c) illustrates the hue shift distortion. The degradation begins subtly in (a) with a slight, unnatural color cast (ΔH=0.05), barely perceptible in the image. In (b), the hue shift becomes more pronounced, reaching a noticeable color alteration (ΔH=0.10). By (c), the effect is most severe, resulting in a complete misrepresentation of the original scene’s colors, which now present a significant deviation from natural hues, disrupting the visual integrity of the image.

The middle row (d, e, f) is dedicated to the saturation shift effect. A slight, noticeable oversaturation is introduced in (d) ($\alpha_{S}=1.2$). This effect intensifies in (e) ($\alpha_{S}=1.4$) and culminates in (f) ($\alpha_{S}=1.6$), where colors appear excessively saturated, creating an almost “cartoon-like” quality with noticeable color bleeding, which severely alters the image’s realism.

The bottom row (g, h, i) demonstrates the intensity gamma distortion (non-linear mismatch), focusing on the impact of over-brightening (γ<1.0). The row begins with (g) (γ=0.4), where a severe brightening effect is observed, making the image appear “washed-out,” with a significant loss of contrast. In (h) (γ=0.6), the image is slightly less washed out, but still lacks the depth and richness of the original. By (i) (γ=0.8), the image shows minimal distortion and is closest to the original contrast, though still exhibiting slight differences in brightness levels. This gamma shift highlights how small adjustments in intensity can cause perceptible distortions in image clarity.

### 3.10. Consistency Analysis of NR and FR Metrics

To quantitatively validate the performance of the proposed NR metric, its scores must be benchmarked against established FR metrics. In this evaluation, the FR metrics (CC, SAM, and SID) are treated as the objective “ground truth” for image quality, as determined in the reduced-resolution validation protocol.

The agreement between the proposed NR metric and these FR metrics is assessed using three standard statistical criteria: the Spearman Rank-Order Correlation Coefficient (SROCC), the Pearson Linear Correlation Coefficient (PLCC), and the Root Mean Square Error (RMSE). These metrics constitute the standard protocol for validating image quality assessment algorithms [41,42] and are extensively employed in evaluating recent remote sensing and multi-focus image fusion frameworks [43,44].

Before calculating the PLCC and RMSE, a non-linear logistic regression is applied to the raw NR metric scores ($u_{i}$) to map them onto the same scale as the FR scores. This results in a mapped predicted score, $y_{i}$. This step is necessary because the raw NR scores and the FR ground truth scores $x_{i}$ may not be linearly related, even if they are monotonically associated.

#### 3.10.1. Pearson Linear Correlation Coefficient (PLCC)

The PLCC measures the prediction accuracy of the NR metric after non-linear mapping. It quantifies the linear correlation between the mapped NR metric scores $y_{i}$ and the FR ground truth scores $x_{i}$. The PLCC is calculated as

(12) $$\mathrm{PLCC}=\frac{\sum_{i=1}^{N}\left(x_{i}-\bar{x}\right)\left(y_{i}-\bar{y}\right)}{\sqrt{\sum_{i=1}^{N}{\left(x_{i}-\bar{x}\right)}^{2}}\sqrt{\sum_{i=1}^{N}{\left(y_{i}-\bar{y}\right)}^{2}}}$$

where $\bar{x}$, $\bar{y}$ are the means of the ground truth scores and the mapped predicted scores, respectively.

#### 3.10.2. Spearman Rank-Order Correlation Coefficient (SROCC)

The SROCC measures the prediction monotonicity of the NR metric. It is a non-parametric test that assesses how well the rank order of the NR scores matches the rank order of the FR scores, without assuming a linear relationship. This is crucial for quality assessment, as a good metric must at least agree on which images are better or worse than others.

The SROCC is calculated as

(13) $$\mathrm{SROCC}=1-\frac{6\sum_{i=1}^{N}{\left(v_{i}-p_{i}\right)}^{2}}{N(N^{2}-1)}$$

where *N* is the total number of fused images; $v_{i}$ is the rank of the ground truth FR score $y_{i}$; $p_{i}$ is the rank of the predicted NR score $u_{i}$.

#### 3.10.3. Root Mean Square Error (RMSE)

The RMSE measures the prediction error. After applying the non-linear logistic mapping, the RMSE quantifies the average magnitude of the error (or residuals) between the mapped NR scores $y_{i}$ and the FR ground truth scores $x_{i}$. The RMSE is calculated as

(14) $$\mathrm{RMSE}=\sqrt{\frac{1}{N}\sum_{i=1}^{N}{\left(x_{i}-y_{i}\right)}^{2}}$$

For a high-performing NR method, the SROCC, PLCC, and KROCC values should be high (closer to one), while the RMSE value should be as low as possible.

### 3.11. Quantitative Validation Results

To quantitatively assess the proposed MVG-SDI, its performance was benchmarked against the competing NR metrics, ${\mathrm{FQNR}}_{\lambda }$ and ${\mathrm{MQNR}}_{\lambda }$. This evaluation was conducted using the RR validation protocol, treating the FR metrics CC, SAM, and SID as the ground truth. The performance was measured using the SROCC for monotonicity, the PLCC for accuracy after non-linear mapping, and the RMSE for prediction error. A superior NR metric should exhibit high SROCC and PLCC values alongside low RMSE values. The results across Table 8, (IK, WV-2) datasets and Table 9, (WV-3, and WV-4) datasets are discussed below, with the best value for each comparison highlighted in red in the corresponding tables.

The proposed MVG method demonstrated consistently strong performance, particularly when benchmarked against the CC and SAM metrics. Against CC, the proposed method achieved the top SROCC, PLCC, and RMSE values on the IK, WV-2, and WV-3 datasets. Against SAM, it secured the best performance across all three correlation criteria (SROCC, PLCC, RMSE) on the IK, WV-2, and WV-3 datasets. While its performance against SID was generally strong (e.g., best SROCC and PLCC on WV-3), the ${\mathrm{MQNR}}_{\lambda }$ metric showed slightly better correlation and lower error against SID on the IK and WV-2 datasets. On the WV-4 dataset, the proposed method performed well but was outperformed by ${\mathrm{FQNR}}_{\lambda }$. Overall, the proposed index proved to be a highly effective and generally consistent metric, especially for predicting CC and SAM.

In contrast, ${\mathrm{FQNR}}_{\lambda }$ showed variable performance. Its SROCC and PLCC values were often lower than the proposed method, especially against CC on the IK (SROCC 0.7308) and WV-3 (PLCC 0.5306) datasets. It exhibited a notably high RMSE (0.3686) when compared against SAM on the WV-2 dataset, indicating significant prediction error in that scenario. However, ${\mathrm{FQNR}}_{\lambda }$ performed exceptionally well on the WV-4 dataset, achieving the best SROCC, PLCC, and RMSE against CC, the best PLCC and RMSE against SAM (tying for SROCC), and tying for the best performance against SID. This indicates ${\mathrm{FQNR}}_{\lambda }$ can be highly accurate under certain conditions but lacks the overall consistency of the proposed method.

${\mathrm{MQNR}}_{\lambda }$ exhibited highly inconsistent performance. It consistently performed extremely well when benchmarked against SID, achieving the top SROCC, PLCC, and RMSE values on the IK and WV-2 datasets. However, its performance against CC and SAM was often poor. Against CC, it yielded very low SROCC and PLCC values on the WV-2 and WV-3 datasets. Against SAM, it produced a high RMSE on the WV-3 (0.4130) and WV-2 (0.3345) datasets, indicating significant prediction errors. Despite these weaknesses, ${\mathrm{MQNR}}_{\lambda }$ performed very strongly on the WV-4 dataset, tying for the best SROCC against CC and SAM. This confirms that ${\mathrm{MQNR}}_{\lambda }$ is highly effective for predicting SID but is unreliable for predicting CC and SAM across different datasets.

### 3.12. Computational Complexity Analysis

To evaluate the practical efficiency of the proposed MVG-SDI, a runtime comparison against three widely used NR metrics, $QNR_{\lambda }$, $MQNR_{\lambda }$, and $FQNR_{\lambda }$, was conducted. The computational complexity of the proposed method is primarily determined by the feature extraction and statistical fitting processes.

Theoretically, the complexity of extracting the FDD and CM features is linear with respect to the number of pixels *N*, i.e., O(N). The subsequent MVG modeling involves calculating the covariance matrix of the feature vectors. With a fixed feature dimension (D=21) and a number of patches *M* proportional to the image size, the fitting process is efficient, scaling as $O(M\cdot D^{2})$. Consequently, the overall computational complexity of the algorithm remains O(N), ensuring it scales linearly with image resolution.

To validate this empirically, the average execution time across the entire IKONOS dataset, which comprises 200 images with a spatial resolution of 1024×1024 pixels, was measured. All experiments were performed on a computer equipped with an Intel Core i5-1035G1 CPU @ 1.19 GHz and 16 GB of RAM, running on a 64-bit operating system. The algorithms were implemented in MATLAB R2024a.

Table 10 presents the average execution times. The results indicate that the proposed MVG-SDI is computationally efficient for practical applications. While it requires slightly more processing time than the simpler $MQNR_{\lambda }$ and $QNR_{\lambda }$ indices due to the statistical modeling involved, it is approximately 35% faster than the advanced $FQNR_{\lambda }$ metric. This demonstrates that the proposed method offers a favorable trade-off, providing sophisticated spectral distortion detection with a runtime comparable to established benchmarks.

## 4. Discussion

### 4.1. Decoupling Spectral Distortion from Spatial Artifacts

The most significant limitation of existing NR metrics, specifically QNR and its variants ($QNR_{\lambda }$, $MQNR_{\lambda }$), is their inability to effectively decouple spectral distortion from spatial information. As evidenced by the visual error maps in Figure 7, Figure 8, Figure 9 and Figure 10, these legacy metrics exhibit a strong “edge bias,” where high distortion scores are concentrated almost exclusively along high-frequency spatial edges (e.g., building boundaries). This suggests that these metrics are conflating spatial sharpening artifacts with spectral fidelity errors, leading to false positives in quality assessment.

In contrast, the proposed MVG-SDI generates error maps that align with the physical surfaces of objects rather than their outlines. By utilizing patch-based statistical fitting rather than pixel-to-pixel differences, the proposed method captures the distributional shift of spectral information. This confirms that the combination of FDD features derived from Benford’s Law and CMs successfully isolates radiometric inconsistencies from spatial sharpening effects, solving a persistent issue in the QNR framework.

### 4.2. Metric Stability and Linearity

For a quality metric to be practically useful in algorithm optimization, it must exhibit monotonicity and linearity, meaning the score should degrade proportionally as the image quality worsens. The degradation simulations in Figure 15, Figure 16, Figure 17 and Figure 18 reveal a critical flaw in the state-of-the-art $FQNR_{\lambda }$ metric: it suffers from premature saturation. In saturation shift experiments, $FQNR_{\lambda }$ spikes to near-maximum error values with even minor degradations ($\alpha_{s}=1.2$), rendering it useless for fine-tuning fusion algorithms as it cannot distinguish between “slightly bad” and “terrible”.

The proposed MVG-SDI, however, demonstrates a consistent, monotonic response across hue, saturation, and non-linear intensity gamma distortions. This linearity is crucial. It implies that the MVG-SDI provides a usable numerical gradient that accurately reflects the magnitude of error.

### 4.3. Generalization and Robustness

A critical advantage of the proposed MVG-SDI is its intrinsic capability to generalize across different sensor types and diverse imaging conditions without requiring retraining or parameter tuning. This robustness stems from the method’s unsupervised, instance-specific learning framework.

Unlike deep learning-based quality metrics that rely on fixed weights learned from a specific training distribution, which thus often suffer from domain shifts when applied to unseen sensors, the proposed method dynamically fits a unique MVG model for every individual image pair. The “ground truth” statistical reference ($\mu_{ref},\Psi_{ref}$) is derived directly from the original MS image of the scene under evaluation. Consequently, the specific spectral response of the sensor (e.g., the four-band configuration of IK vs. the eight-band configuration of WV-2) and the specific imaging conditions (e.g., atmospheric haze, solar angle, or seasonal vegetation changes) are automatically incorporated into the reference model.

Furthermore, the feature set employed, combining FDD based on Benford’s Law and CMs, captures fundamental statistical regularities of natural scenes rather than sensor-specific artifacts. The validity of this approach is empirically supported by the experimental results presented in Section 3, where the metric demonstrated consistent performance across four distinct satellite sensors (IK, WV-2, WV-3, and WV-4) covering varying spatial resolutions and spectral band configurations.

### 4.4. Discrepancies Between NR and FR Metrics

While Table 8 and Table 9 demonstrate strong overall correlations between MVG-SDI and FR metrics (e.g., SROCC up to 0.9000 with CC on WV-3), specific ranking disagreements in Table 4, Table 5, Table 6 and Table 7 raise questions about their origins. For example, on the WV-3 dataset, the MVG-SDI identifies GS as the best performer but flags BT-H (strong in SID) as the worst; on WV-4, it ranks PWMBF highest despite its suboptimal SAM. These discrepancies do not necessarily indicate a limitation of the MVG-SDI but rather highlight inherent constraints in the FR protocol itself. Wald’s RR protocol, while a standard benchmark, relies on downscaling assumptions that may not fully capture real-world FR distortions. Issues such as scale-invariance failures at high resolution ratios, biases from MTF filters, and sensor aging effects (as noted in prior work [17]) can lead FR metrics to over- or under-penalize certain algorithms, particularly those introducing non-linear spectral changes not evident at reduced scales. In contrast, the MVG-SDI’s NR design, focusing on multivariate statistical deviations (e.g., Mahalanobis distance between patch-based FDD and CM features), detects global and localized spectral inconsistencies without these scaling artifacts, making it more robust to such biases. That said, the MVG-SDI’s exclusive emphasis on spectral distortion (as a deliberate design choice) may contribute to disagreements in cases where spatial artifacts indirectly influence spectral statistics, though this is mitigated by its patch-based approach and decoupling from edge-biased features (Section 4.1). Ultimately, these divergences suggest that FR protocols, while valuable for validation, have limitations in generalizing to operational scenarios without a ground truth—positioning the MVG-SDI as a complementary tool that addresses these gaps rather than a flawed alternative.

### 4.5. Limitations and Future Research

#### 4.5.1. Limitations

The primary limitation of the proposed MVG-SDI is its exclusive focus on spectral fidelity. As indicated by its design, the feature extraction process utilizing Benford’s Law on angular components and Color Moments is engineered strictly to isolate radiometric inconsistencies and color shifts. While this effectively solves the issue of spectral–spatial conflation found in legacy metrics like QNR, it renders the index blind to spatial distortions. Consequently, the MVG-SDI cannot detect structural degradations such as blurring or ghosting artifacts. For a holistic quality assessment, this index must currently be paired with a separate, dedicated spatial quality metric.

#### 4.5.2. Future Work

Future research will focus on three strategic directions to maximize the utility of the MVG framework and advance the standardization of NR assessment:

Unified Quality Framework:

The immediate goal is to develop a comprehensive Multivariate Gaussian framework capable of quantifying both spectral and spatial distortions within a single model. We aim to construct a complementary MVG-based spatial distortion index utilizing features such as gradient profile sharpness and Laplacian statistics. The critical challenge will be integrating these features without re-introducing the “coupling” effects that plague current benchmarks. By combining this future spatial index with the current MVG-SDI, we intend to create a holistic “MVG-QNR” metric that maintains the statistical robustness demonstrated in this work, specifically the stability against hue and saturation shifts while independently penalizing spatial errors.

Loss Function for Deep Learning: Given that the MVG-SDI relies on differentiable statistical operations, a promising avenue is to integrate it as a perceptual loss function for training pansharpening CNNs. Unlike standard $L_{1}$ or $L_{2}$ losses that minimize pixel-wise errors, an MVG-SDI-based loss would force the network to explicitly prioritize the preservation of statistical spectral distributions, potentially mitigating the spectral color shifts often observed in deep learning-based fusion.

Extension to Hyperspectral and Multi-modal Imaging: While the current MVG-SDI is designed for pansharpening, the underlying multivariate statistical framework shows promise for other fusion tasks, such as infrared and visible image fusion. Specifically, the statistical foundation of this method, FDD in the HCS, is theoretically scalable to higher-dimensional data. Future work will investigate adapting the feature set to capture the specific statistical characteristics of hyperspectral pansharpening and thermal–visible fusion. In these domains, the preservation of precise radiometric signatures is paramount for downstream tasks such as material identification and classification.

## 5. Conclusions

This paper presents the No-Reference Multivariate Gaussian-based Spectral Distortion Index (MVG-SDI), a specialized metric for evaluating spectral distortions in pansharpened remote sensing images. By overcoming limitations in existing NR methods, such as conflating spectral and spatial artifacts, the MVG-SDI isolates spectral fidelity through patch-based analysis, combining FDD features from Benford’s Law in HCS and CM features. These are fitted to MVG models for the original MS and fused images, with distortion measured via Mahalanobis distance.

Experiments on the NBU dataset across five sensors (IKONOS, WorldView-2/3/4) show the MVG-SDI outperforming FQNR_*λ*_ and MQNR_*λ*_ in correlations with FR metrics like CC, SAM, and SID. It achieves high SROCC and PLCC values (e.g., 0.8089 SROCC and 0.9719 PLCC against CC on IK), with robust sensitivity to simulated distortions like hue and saturation shifts.

## Figures and Tables

**Table 1:** Details of the NBU dataset used for validation.

Sensor	Image Pairs	PAN Dimensions	MS Dimensions	MS Bands
IK	200	1024×1024 pixels	256×256 pixels	4
WV-2	500	1024×1024 pixels	256×256 pixels	8
WV-3	160	1024×1024 pixels	256×256 pixels	8
WV-4	500	1024×1024 pixels	256×256 pixels	4

**Table 2:** Summary of typical pansharpening methods used for quality assessment.

Category	Fusion Method	Description
CS	BT-H	Brovey transform with haze correction [17]
	BDSD	Band-dependent spatial-detail-based [9]
	BDSD-PC	BDSD with physical constraints [10]
	GS	Gam–Schmidt [7]
	PRACS	Partial replacement adaptive CS [20]
MRA	AWLP	Additive wavelet luminance proportional [11]
	C-MTF-GLP-CBD	MTF-GLP-CBD [21,22,23] with local parameter estimation-exploiting clustering [24]
	MTF-GLP	GLP with modulation transfer function-matched filter [12]
	MTF-GLP-CBD	MTF-GLP [21,22] context-based decision with regression-based injection model [23]
	MTF-GLP-FS	MTF-GLP, with a full-scale regression-based injection model [40]
	MTF-GLP-HPM	MTF-GLP high-pass modulation injection model [15]
	MTF-GLP-HPM-H	MTF-GLP-HPM, with haze correction [16]
	MTF-GLP-HPM-R	MTF-GLP-HPM [21,25] with preliminary regression-based spectral matching phase [26]
	MF	Non-linear decomposition scheme exploiting half-gradient morphological filters [27]
VO	FE-HPM	Filter estimation based on a semi-blind deconvolution framework and HPM injection model [28]
	SR-D	Sparse representation of injected Details [14]
	PWMBF	Principal component analysis/wavelet model-based fusion [18]
	TV	Total variation pansharpening [29]
ML	PNN	Pansharpening neural network [19]
	PNN-IDX	PNN with input auxiliary indexes [19]
	A-PNN	Advanced PNN [19]
	A-PNN-FT	A-PNN with fine tuning [19]

**Table 3:** Fixed parameters and implementation settings for MVG-SDI.

Parameter	Value	Description
Patch size	32×32	Size of non-overlapping blocks for local feature extraction.
HCS normalization	2/π	Constant used to normalize angular components $\theta_{k}$ to [0,1].
FDD bins	9	Digits {1,…,9} used for First Digit Distribution analysis.
CM features	12	Mean, std. dev, and skewness calculated for 4 channels (R, G, B, NIR).
FDD features	9	Probability of leading digits derived from HCS angular components.
Total dimensionality	21	Concatenated feature vector size per patch.

**Table 4:** The numerical evaluation results on the IK dataset (best results in red, worst in blue).

Category	Fusion Model	FR Evaluation			NR Evaluation			
		CC	SAM	SID	QNR_*λ*_	MQNR_*λ*_	FQNR_*λ*_	Ours
CS	BT-H	0.9366	4.0570	0.0081	0.0930	3.2136	0.3108	0.1320
	BDSD	0.9296	4.2885	0.0095	0.0630	1.9446	0.0616	0.1001
	BDSD-PC	0.9297	4.2824	0.0094	0.0387	1.9316	0.0611	0.0986
	GS	0.9005	4.5496	0.0102	0.0439	1.8151	0.1346	0.0442
	PRACS	0.9105	4.3131	0.0090	0.0384	2.6681	0.0436	0.0365
MRA	AWLP	0.9105	4.3131	0.0090	0.1288	2.9369	0.0250	0.1146
	C-MTF-GLP-CBD	0.9333	4.0767	0.0085	0.0872	2.4064	0.0271	0.0834
	MTF-GLP	0.9246	4.3186	0.0095	0.1465	2.8717	0.0306	0.1330
	MTF-GLP-CBD	0.9275	4.2574	0.0090	0.0980	2.5289	0.0282	0.0979
	MTF-GLP-FS	0.9285	4.2224	0.0089	0.1027	2.5313	0.0283	0.1015
	MTF-GLP-HPM	0.9306	4.0504	0.0083	0.1425	2.9444	0.0286	0.1314
	MTF-GLP-HPM-H	0.9366	4.0601	0.0081	0.1145	3.3280	0.0262	0.1031
	MTF-GLP-HPM-R	0.9341	3.9898	0.0081	0.0969	2.3714	0.0265	0.0975
	MF	0.9285	4.0357	0.0083	0.1225	2.9477	0.0356	0.1126
VO	FE-HPM	0.9345	4.0220	0.0082	0.1134	2.8001	0.0295	0.0914
	SR-D	0.9284	4.0172	0.0083	0.1008	2.2626	0.0138	0.1466
	PWMBF	0.9129	4.6427	0.0107	0.1621	2.5900	0.0982	0.0789
	TV	0.9355	3.8442	0.0079	0.0305	2.4211	0.0419	0.0799
ML	PNN	0.9509	3.3943	0.0062	0.0195	2.3445	0.0225	0.1017
	PNN-IDX	0.9508	3.4613	0.0064	0.0275	2.6387	0.0233	0.1028
	A-PNN	0.9505	3.3978	0.0064	0.0056	2.2108	0.0216	0.1027
	A-PNN-FT	0.9344	3.7313	0.0073	0.0103	2.2794	0.0215	0.1059

**Table 5:** The numerical evaluation results on the WV-2 dataset (best results in red, worst in blue).

Category	Fusion Model	FR Evaluation			NR Evaluation			
		CC	SAM	SID	QNR_*λ*_	MQNR_*λ*_	FQNR_*λ*_	Ours
CS	BT-H	0.9476	6.5261	0.0323	0.0075	2.0961	0.1981	0.0655
	BDSD	0.9362	7.6800	0.0513	0.0453	3.8600	0.2084	0.0895
	BDSD-PC	0.9434	7.2859	0.0453	0.0174	3.2862	0.1545	0.0398
	GS	0.9137	7.5721	0.0432	0.0172	1.9843	0.0157	0.0258
	PRACS	0.9215	8.3073	0.0529	0.0189	4.0798	0.0613	0.0395
MRA	AWLP	0.9215	8.3073	0.0529	0.0554	5.4484	0.0222	0.1054
	C-MTF-GLP-CBD	0.9370	7.1024	0.0437	0.0334	2.9036	0.0279	0.0539
	MTF-GLP	0.9458	6.7286	0.0369	0.0696	3.4910	0.0245	0.0942
	MTF-GLP-CBD	0.9382	7.3092	0.0438	0.0579	3.4599	0.0249	0.0871
	MTF-GLP-FS	0.9395	7.2155	0.0428	0.0604	3.4824	0.0248	0.0905
	MTF-GLP-HPM	0.9467	6.8251	0.0369	0.0653	3.0096	0.0250	0.1002
	MTF-GLP-HPM-H	0.9479	6.4964	0.0320	0.0703	3.6573	0.0246	0.1003
	MTF-GLP-HPM-R	0.9387	7.4596	0.0447	0.0548	3.4259	0.0255	0.0936
	MF	0.9093	7.2319	0.0400	0.0662	3.2228	0.0317	0.1050
VO	FE-HPM	0.9183	7.2296	0.0401	0.0621	3.0923	0.0270	0.0999
	SR-D	0.9257	6.8961	0.0387	0.0492	2.7420	0.0373	0.1506
	PWMBF	0.9322	7.5824	0.0447	0.1018	3.2240	0.0644	0.0955
	TV	0.9444	6.5577	0.0363	0.0180	4.0350	0.0337	0.1053
ML	PNN	0.9444	6.0717	0.0264	0.0607	5.3151	0.0489	0.0369
	PNN-IDX	0.9452	5.9437	0.0262	0.0797	5.7830	0.0520	0.0373
	A-PNN	0.9409	6.1417	0.0280	0.0687	4.8982	0.0425	0.0448
	A-PNN-FT	0.9332	6.3961	0.0318	0.0546	4.1734	0.0457	0.0587

**Table 6:** The numerical evaluation results on the WV-3 dataset (best results in red, worst in blue).

Category	Fusion Model	FR Evaluation			NR Evaluation			
		CC	SAM	SID	QNR_*λ*_	MQNR_*λ*_	FQNR_*λ*_	Ours
CS	BT-H	0.9488	6.3993	0.0316	0.0175	1.7406	0.4923	0.2899
	BDSD	0.9493	6.7784	0.0405	0.0318	2.9233	0.1881	0.0989
	BDSD-PC	0.9504	6.6868	0.0378	0.0567	2.1396	0.1762	0.0852
	GS	0.9381	7.6466	0.0409	0.0275	1.9514	0.0225	0.0243
	PRACS	0.9436	6.8305	0.0391	0.0244	2.6945	0.1000	0.0316
MRA	AWLP	0.9436	6.8305	0.0391	0.0721	2.8280	0.0372	0.0981
	C-MTF-GLP-CBD	0.9442	6.9864	0.0424	0.0401	2.6732	0.0465	0.0485
	MTF-GLP	0.9507	6.4342	0.0330	0.0983	2.9332	0.0426	0.0917
	MTF-GLP-CBD	0.9498	6.4429	0.0338	0.0797	2.6882	0.0429	0.0785
	MTF-GLP-FS	0.9498	6.4494	0.0340	0.0831	2.7234	0.0428	0.0825
	MTF-GLP-HPM	0.9448	6.4866	0.0360	0.0866	2.8600	0.0446	0.1140
	MTF-GLP-HPM-H	0.9497	6.3894	0.0319	0.1091	3.3463	0.0421	0.1075
	MTF-GLP-HPM-R	0.9486	6.6016	0.0372	0.0665	2.7434	0.0443	0.0994
	MF	0.9370	6.6055	0.0361	0.0917	2.9459	0.0427	0.0992
VO	FE-HPM	0.9447	6.5691	0.0360	0.0843	2.8014	0.0425	0.0982
	SR-D	0.9381	6.7831	0.0668	0.0689	2.2704	0.0251	0.1525
	PWMBF	0.9403	7.2251	0.0403	0.1579	3.6927	0.0939	0.1069
	TV	0.9485	6.7804	0.0489	0.0249	2.9708	0.0458	0.1308
ML	PNN	0.9248	7.5878	0.0426	0.0490	3.6573	0.0831	0.1112
	PNN-IDX	0.9048	9.2221	0.0966	0.0615	4.6988	0.1403	0.1358
	A-PNN	0.9207	6.8288	0.0407	0.0696	3.9070	0.0571	0.1146
	A-PNN-FT	0.9444	6.3169	0.0353	0.0507	3.7100	0.0672	0.0857

**Table 7:** The numerical evaluation results on the WV-4 dataset (best results in red, worst in blue).

Category	Fusion Model	FR Evaluation			NR Evaluation			
		CC	SAM	SID	QNR_*λ*_	MQNR_*λ*_	FQNR_*λ*_	Ours
CS	BT-H	0.9558	3.7117	0.0083	0.0894	2.2341	0.3840	0.1517
	BDSD	0.9582	3.8653	0.0089	0.0174	1.6269	0.1075	0.0363
	BDSD-PC	0.9585	3.8471	0.0088	0.0174	1.6269	0.1075	0.0363
	GS	0.9571	3.7687	0.0082	0.0359	1.7188	0.1549	0.0286
	PRACS	0.9629	3.9529	0.0092	0.0264	1.5953	0.0859	0.0205
MRA	AWLP	0.9629	3.9529	0.0092	0.0370	1.7995	0.0293	0.0361
	C-MTF-GLP-CBD	0.9596	3.9070	0.0096	0.0359	1.9339	0.0310	0.0188
	MTF-GLP	0.9609	3.6775	0.0090	0.0619	1.9296	0.0326	0.0397
	MTF-GLP-CBD	0.9628	3.8338	0.0090	0.0586	1.9327	0.0319	0.0344
	MTF-GLP-FS	0.9625	3.8248	0.0090	0.0585	1.9313	0.0319	0.0344
	MTF-GLP-HPM	0.9615	3.6997	0.0080	0.0609	2.0237	0.0327	0.0391
	MTF-GLP-HPM-H	0.9571	3.8501	0.0090	0.0364	1.7022	0.0326	0.0466
	MTF-GLP-HPM-R	0.9626	3.9861	0.0094	0.0565	2.0015	0.0320	0.0348
	MF	0.9618	3.6794	0.0079	0.0674	2.1406	0.0371	0.0433
VO	FE-HPM	0.9659	3.6680	0.0078	0.0587	2.0166	0.0307	0.0380
	SR-D	0.9585	3.9080	0.0212	0.0256	1.6037	0.0144	0.0680
	PWMBF	0.9584	4.4891	0.0109	0.0612	2.0441	0.0812	0.0136
	TV	0.9628	4.0023	0.0095	0.0279	1.9946	0.0383	0.0288

**Table 8:** Consistency analysis results of NR and FR metrics on the IK and WV-2 datasets (best results are highlighted in red).

FR Metric	NR Metric	IK			WV-2		
		SROCC	PLCC	RMSE	SROCC	PLCC	RMSE
CC	Ours	0.8089	0.9719	0.0032	0.7000	0.9594	0.0036
	FQNR_*λ*_	0.7308	0.8602	0.0113	0.6000	0.8791	0.0951
	MQNR_*λ*_	0.6070	0.5602	0.0113	0.5000	0.0022	0.0129
SAM	Ours	0.8903	0.8765	0.0752	0.8801	0.9510	0.0095
	FQNR_*λ*_	0.8810	0.7720	0.0993	0.8605	0.7718	0.3686
	MQNR_*λ*_	0.6000	0.7933	0.0951	0.7000	0.8168	0.3345
SID	Ours	0.7800	0.8326	0.0004	0.8704	0.8313	0.0066
	FQNR_*λ*_	0.8700	0.8276	0.0004	0.8505	0.7412	0.0061
	MQNR_*λ*_	0.9000	0.9369	0.0002	0.9000	0.8800	0.0035

**Table 9:** Consistency analysis results of NR and FR metrics on the WV-3 and WV-4 datasets (best results are highlighted in red).

FR Metric	NR Metric	WV-3			WV-4		
		SROCC	PLCC	RMSE	SROCC	PLCC	RMSE
CC	Ours	0.9000	0.9937	0.0005	0.6669	0.9183	0.0009
	FQNR_*λ*_	0.8000	0.5306	0.0039	0.9747	0.9878	0.0004
	MQNR_*λ*_	0.3000	0.4301	0.0046	0.9747	0.5610	0.0024
SAM	Ours	0.9000	0.9839	0.1251	0.6669	0.8017	0.0496
	FQNR_*λ*_	0.8000	0.9628	0.3444	0.9747	0.9968	0.0066
	MQNR_*λ*_	0.3000	0.1331	0.4130	**0.9747**	0.9929	0.0098
SID	Ours	0.7800	0.9484	0.0011	0.7591	0.8909	0.0003
	FQNR_*λ*_	0.7000	0.9459	0.0011	0.8751	0.9939	0.0001
	MQNR_*λ*_	0.4000	0.9459	0.0011	0.8721	0.9929	0.0002

**Table 10:** Average execution time (in seconds) on the IKONOS dataset.

Metric	Average Time (s)
MQNR_*λ*_	0.6898
QNR_*λ*_	1.1077
Ours	1.9955
FQNR_*λ*_	3.0951

## Data Availability

Publicly available datasets were analyzed in this study. This data can be found in [34].
